# Explainability and importance estimate of time series classifier via embedded neural network

**DOI:** 10.1038/s41598-025-17703-w

**Published:** 2025-10-03

**Authors:** Ho Tung Jeremy Chan, Ilija Šimić, Eduardo Veas

**Affiliations:** 1 Institute of Human-Centred Computing, Graz University of Technology, Graz, 8010 Austria; 2 Human-AI Interaction, Know Center Research GmbH, Graz, 8010 Austria

**Keywords:** Computer science, Scientific data

## Abstract

Time series is common across disciplines, however the analysis of time series is not trivial due to inter- and intra-relationships between ordered data sequences. This imposes limitation upon the interpretation and importance estimate of the features within a time series. In the case of multivariate time series, these features are the individual time series and the time steps, which are intertwined. There exist many time series analyses, such as Autocorrelation and Granger Causality, which are based on statistic or econometric approaches. However analyses that can inform the importance of features within a time series are uncommon, especially with methods that utilise embedded methods of neural network (NN). We approach this problem by expanding upon our previous work, Pairwise Importance Estimate Extension (PIEE). We made adaptations toward the existing method to make it compatible with time series. This led to the formulation of aggregated Hadamard product, which can produce an importance estimate for each time point within a multivariate time series. This subsequently allows each time series within a multivariate time series to be interpreted as well. Within this work, we conducted an empirical study with univariate and multivariate time series, where we compared interpretation and importance estimate of features from existing embedded NN approaches, an explainable AI (xAI) approach, and our adapted PIEE approach. We verified interpretation and importance estimate via ground truth or existing domain knowledge when it is available. Otherwise, we conducted an ablation study by retraining the model with Leave-One-Out and Singleton feature subsets to see their contribution towards model performance. Our adapted PIEE method was able to produce various feature importance heatmaps and rankings inline with the ground truth, the existing domain knowledge or the ablation study.

## Introduction

Deep learning (DL) refers to algorithms that use a cascade of computational units (neurons) to learn hierarchical representations of the data in the form of a neural network (NN) model. This enables NNs to identify relevant data aspects within the data and feature select internally without the need of manual feature engineering^1^. However, there also exists issues within the internal feature selection (FS). Although a NN model is connected by computational units, the internal working between the connections is often complex and obscured by non-linearity. This makes it difficult to explain how the output is derived from the input given to the NN, specifically which part of the input is meaningful to the derived output. However, the identification of important features within the input can provide informative details that could be useful in understanding the dataset and improving the existing model. For example, if a model is being used in some application of some industry, e.g medical or finance. Being able to understand what features such a model relies on for its predictions enables to verify the model and improving its performance^2^.

There exists many NN explainability methods^3,4^ and NN FS methods for feature datasets. Feature datasets are datasets which consist of unordered features, $\{f\}$. However, NN explainability methods^5,6^ and existing NN FS methods for time series are scarce when compared to other forms of data, such as explainability with images^7^. This is often due to methods not being transferable towards time series, or they have not been validated within a time series context. *Time series* refers to datasets with features ordered in time as a sequence, (*f*). Time series can have one (univariate), $\{(f)\}$, or multiple (multivariate) channels, $\{(f_1), (f_2),...\}$. Time series data is prevalent and determinant for decisions in domains such as medical, financial and industrial. A common use of time series is the analysis of variation over time to identify patterns in order to make informed decisions^8^. It is common in time series data to be multivariate, which creates a high dimensional feature space^9^. In such cases, knowing what features are important can reduce the computational complexity in training. This also helps to develop a better understanding of the data.

Just as dimension reduction can assist in understanding feature datasets, it can also assist in understanding time series. In the domain of dimension reduction, feature transformation and feature selection are different approaches. Feature transformation with unordered features (feature dataset domain) focuses on transforming the data into a lower dimension with minimal loss to the significance present within the data. Common feature transformation includes, PCA^10^, t-SNE^11^, etc. However, in time series signal processing, feature transformation does not only consist of domain reduction. There exists other approaches such as Fourier Transform^12^ and Wavelet Transform^13^, which performs feature transformation upon the signal by focusing on the information in the frequency domain instead of the time domain. There also exist a topological feature transformation approach^14^ using persistant landscape. On the other hand, FS refers to techniques that select a subset of features from the available feature space and discard the rest. This retains semantic meaning within the chosen data whilst avoiding the loss of information from the transformation of dimensions. FS with time series are however more complicated. This is due to the inter-connection between the features from the increase in dimensions, where the removal of a feature from one dimension could inadvertently affect another feature from the other dimension. This challenge of FS within time series context is not well studied, at least when it comes to how the features from each dimension affect one another. This inadvertently means that the applicability of FS methods within time series context is questionable when it comes to selecting features or removing features from time series.

There are other ways to understand time series. There exist time series analysis and econometric approaches. Cross Correlation measures the displacement between 2 time series, and can be used as a similarity measure. Autocorrelation^15^ identifies repeating patterns within a time series. Granger Causality^16^ expand upon such and tests for predictive causation between time series. However, despite the number of existing time series analysis based on statistic and econometric, little of these methods explicitly identify what features are important within the time series. They simply implicitly employ the use of these components in their outcome or output. In terms of machine learning methods for time series, there are existing methods such as Shapley Values^17^, Shapley Additive Global importancE^18^ (SAGE), and Random Forest, which are often limited to univariate series and cannot be easily applied to multivariate series without increasing computational complexity. For example, in the approaches of DeepSHAP^19^, GradientExpaliner^20^ using integrated gradients, and the TreeExplainer^21^. Although it is possible to apply such methods to univariate time series, they do not necessarily transfer to multivariate time series, or at least it is not possible without heavy modifications towards the existing mechanisms. In which case, it is no longer the same method but something else entirely.

Previously, we have proposed a form of analysis for NNs that can estimate the relative importance of features from a dataset, which we named Pairwise Importance Estiamte Extension^22^ (PIEE). This is accomplished by embedding a pairwise layer of weights in between the input and the first layer of a model. When the model is trained, its internal feature selection can be reflected upon the embedded pairwise layer of weights through back propagation, which can be analysed to determine the relevance between the features of the dataset through the hierarchy principle^23,24^ that is studied in statistics. In this work, we expand upon PIEE’s estimate of feature importance by showing that it is applicable with univariate time series, and it can be further adapted towards multivariate time series via some engineering based on the principles of matrix multiplication upon the essential pairwise layer and adjusting the necessary analysis. We showcase how the importance of individual time series within a multivariate time series can be determined via element wise multiplication with an embedded pairwise layer, and how the importance estimate can be used for analysis. We also show how the importance of time steps of each individual time series within a multivariate time series can be defined via the Hadamard product with an embedded pairwise layer, and how the importance estimate can be used for analysis. Additionally, we show how the pairwise layer’s results from the Hadamard product of each time step can be aggregated to produce an overall importance estimate for their corresponding individual time series within the context of a multivariate time series.

Despite the necessary adaptation of PIEE for time series, PIEE remains generalisable to NNs with minimal complexity, and easy to interpret. Simulated datasets and real applied datasets were used to demonstrate the intricacy of the modifications towards the PIEE’s methods. Any estimated significance from the analyses can be confirmed with the ground truth from the simulated cases, however this information or the knowledge from experts is not always available in real applied datasets. In such cases, we verified identified significance and redundancy via an ablation study that combines Sensitivity Analysis^25^ and Reduce and Retrain^26^. Additionally, we verified PIEE’s methods by comparing them with a well-established eXplainable AI method (xAI), specifically DeepLIFT^27^.

Within this work, we demonstrate PIEE with several case studies concerning different datasets and different NN architecture. They include a multiclass classification simulated univariate dataset and multivariate dataset from Section E1: Simulated Time Series Datasets, a binary classification of error decoding via EEG signals from Section E2: AR EEG Error Decoding, a binary classification multivariate occupancy detection time series from Section E3: Occupancy Detection, and a multiclass classification multivariate activity recognition time series from Section E4: HHAR. We compare the adapted PIEE’s methods with a variety of existing NN related methods. This includes existing embedded FS NN methods: Deep Feature Selection^28^ (DF) and NeuralFS^29^ (NFS), as well as an xAI method, DeepLIFT^27^. In the comparison, we showed some of the existing embedded FS NN’s disadvantages with respect to time series, as well as highlighted the consistency of PIEE even when applied to time series. We used the ground truth for verification when possible in Section Verifying Importance via Ground Truth, where we compared the visualisation of identified feature importance. We also conducted an ablation study by combining established verification procedures, such as Sensitivity Analysis and Reudce and Retrain, to further verify importance estimate when the ground truth is not available in Section Verifying Importance via Ablation Study. Specifically, we retrained models with Leave-One-Out and Singleton feature subsets, and measured the deviation between performances to verify the contribution of each feature as per the principal of ablation study. Lastly, we performed a comparison of F1-Score between the different method’s classifier as an additional measure of performance between the different methods in Section Performance Comparison.

## Background

### Neural Networks

Neural networks are computational models that consist of *L* layers of neurons connected in a sequence. An example is available in Fig. 4(a). Each layer $l_j, j\in 1,\ldots ,L$ is a parametric function acting on the output of the previous layer such that the network is a chain of composed functions optimised to perform a task. A layer contains neurons, which are small computational units that perform transformation upon the data. The activation of a neuron is controlled by a weight and a bias, which respectively represent the strength of the link to the previous layer and the deviation from the intended output^30^. The output of each layer $l_j$ constitutes a representation of the input domain $X_{j}$^31^. In order to issue a prediction *O* the NN performs a forward pass, $O= f(X_0)=f_L(W_L,f_{L-1})$

The collection of weights $W_j$ and bias $b_j$ of a layer $l_j$ are trainable parameters. We will assume no bias term for simplicity and retain $W_j \in W$ as the set of trainable parameters of layer $l_j$, where *W* is the set of all trainable parameters in the network. At the start, the parameters of the network $W=\{W_1, \ldots , W_L\}$ are randomly initialised. During the training phase^32^, a dataset *D* with known input-output pairs $(X_i, Y_i)$ is used so that the network attempts to predict the output and any deviation is corrected by adjusting the parameters *W*. To do so, the output is first computed through forward propagation, followed by a cost function *E* that measures the discrepancy between the prediction $O_i$ and the known outcome $Y_i$, the *prediction loss*. Using the partial derivative of *E* with respect $f_L$, it is possible to compute the partial derivatives of *E* with respect to $W_L$ and $f_{L-1}$ by applying the derivative chain rule with backward recurrence. This reverse-mode differentiation method computes the derivative of *E* with respect to every node.

The goal during training is to iteratively adapt the parameters *W* in order to minimise *E*. The minimisation procedure adjusts the weights while training by using a learning rate $\eta$ in a *gradient descent algorithm*, $W(z) = W(z-1)- \eta \frac{\partial {E}}{\partial {W}}$, where *z* is the layer of the NN.

### Time Series Modelling

Time series data is a sequence of real values indexed at successive time intervals $x_{(t)}=\{(x_1,t_1), \ldots ,(x_n,t_n)\}$, which is frequently referred to as a Random variable sequence $x_{(t)}=(x_1, \ldots ,x_n)$. An univariate time series *U* consists of a single sequence $x_{(t)} \in \mathbb {R}^{n}$, whereas a *c*-dimensional multivariate time series consists of *c* univariate time series $(U_1,\ldots ,U_c)\in \mathbb {R}^{c \times n}$.

A time series model assumes a relationship associating an output with some input time series, which can be univariate or multivariate. It achieves this by using inter- or intra- relationship within the sequence or sequences. Time series can be defined as stationary or non-stationary^33^. Stationary time series is a series where observable values are not impacted by time related factors, such as trends, seasonality, etc. And vice versa for non-stationary time series. Within time series analysis, Cointegration^33^ is a method of time series modelling that linearly combines different drifted version of the non-stationary time series to form a stationary time series. Another common time series model is time series regression. Consider a multivariate time series which contains different univariate time series *x*1, *x*2. Together they can formulate a time series regression model $y_t=x1_{(t)}\beta + x2_{(t)}\theta$. Time series regression is often used for forecasting, and the staple of time series forecasting in time series analysis and econometric approaches is the Auto Regressive Integrated Moving Average^34^ (ARIMA) approach, which has many variations. ARIMA utilises time series decomposition to identify trend and seasonality, in order to anticipate and adjust for fluctuation accordingly when forecasting.

Aside from time series regression, there also exists time series classification. In a classification problem, some event will be observable at different times. The events can have a *K* number of variations (*K* number of classes) which can be defined by one hot encoding. Time series classification^9^ aims to train a classifier on dataset *D* to map the input to a probability distribution over the one hot encoded label. A time series can be split into windows with the sliding window approach in order to capture such an event. The sliding windows can overlap or not depending on the offset, also known as stride.

The traditional architecture for NNs, the fully connected network or the multi-layer perceptron, has difficulty when it comes to modelling the temporal dependency within the time steps of a time series. There exists a branch of NN architecture, named Recurrent neural networks (RNN), which are frequently used to model time series data. This is due to its reuse of the activations from the feed forward pass to model the temporal dependency, and causal relationships within the time series^35^. RNNs are however difficult to parallelize and suffer from the vanishing gradient problem, especially with complex time series^9^. In terms of RNNs, Long-Short-Term-Memory^36^ (LSTM) is better at dealing with long sequences, as it utilizes the concept of memory cell and gate units to make it less prone to vanishing gradient during backpropagation. It has been shown in multiple cases^37,38^ of time series forecasting that the performance of advance NN time series models are comparable, if not better, than time series models following time series analysis and econometric approaches. In Khorshid, et al.^39^, it has been found that NN forecasting models are capable of multi-modal and non-linear modelling with regards to optimisation and prediction, whereas ARIMA is limited to predominantly linear modelling of univariate time series. This highlights the significance of NN time series models and necessitates the understanding of such models.

### Feature Selection and Feature Importance

A feature is a property of the data that is measurable. Feature selection (FS) is a process which aims to select a smaller subset of features, $D*$, from the complete set of features, *D*, to perform the task at hand. There are 3 main types of FS methods: Filter^40^, Wrapper^41^, and Embedded^42^. Each follows a different principal to select a subset of features that could produce the best performance.

PIEE^22^ follows the principals of embedded methods, which are methods that adapt the subset of features for selection as the model trains. A notable difference is that the proposed method does not utilise feature sparsity within hierarchy constraints^43,44^ to arrive at the subset of features for selection, even though this tends to be the norm for embedded NN FS methods.

Feature importance estimation aims to assign a score $s_i\in \mathbb {R}$ to each feature $x_i$ that quantifies the significance of the feature with regards to the response of the model. An importance rating of each feature is formulated as follows $s=(s_1,\ldots ,s_F)\in \mathbb {R}$ where *F* is the number of features. This quantifies the significance of each feature from $x_i$ in computing and affecting the outcome. The feature importance rating of each feature allows for FS through enforcing some defined threshold, where all selected features should have a score higher than the threshold. This is to ensure that only features with a strong response are considered for a new dataset $D^*$.

In the context of time series, additional complexity will need to be considered, such as the causal relationship within an univariate time series, as well as possible ties with other time series within the dataset. In the case of univariate time series $x_{(t)} \in \mathbb {R}^n$, we seek after the quantification of relative feature importance as before. However, the distinction is that each feature, the time steps, in an univariate time series is ordered thus there is an extra layer of complexity due to the effects of causality.

In a multivariate time series, a feature can be the channels of univariate time series or the time steps. Similar to before, it is assumed that a dataset $D^*$ can be extracted from *D* which retains the desired information of causality, and capture any relevant relationship between time series for multivariate time series. In the case of multivariate time series, not only are there inter-relationship within each time series, there are also intra-relationships between each time series. For example, an accelerometer will have a time series for the x-axis, y-axis, and z-axis; where each time series will have some dependency with the others as they measure the same movement in different axis.

There exist various approaches for estimating feature importance, such as approaches that rely on the models gradients^45,46^, information backpropagation^27,47^, feature occlusion^48^, surrogate models^49^ or game theory^50^. Depending on the scope of the feature importance estimate, they can be grouped into global or local approaches^51^. Global feature importance approaches identify the general decision-making strategies of the model, whereas local approaches estimate the feature importance for single samples. Moreover, feature importance estimates can be model-agnostic, which can be used with any model type, or model-specific which are tailored to specific models. PIEE falls into the category of global, model-specific methods. While it is limited to NNs, it can be easily transferred and applied to any kind of NN.

**Fig. 1:**
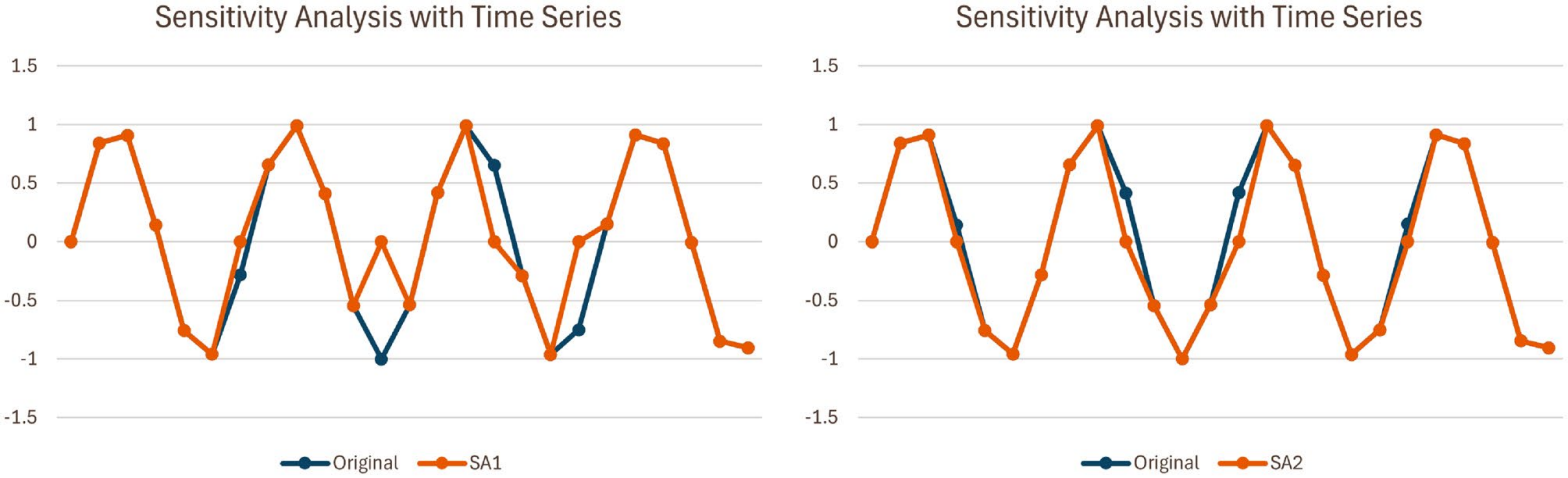
Sensitivity Analysis with Perturbation within the context of time series, where different parts of the series are replaced with 0.

**Fig. 2:**
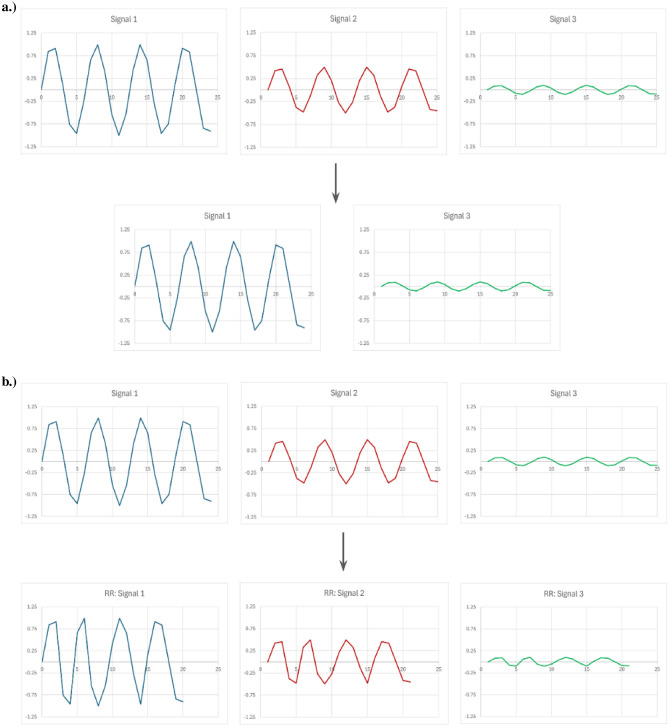
Reduce and Retrain within the context of multivariate time series where signals from the time series are removed. **b.)** Reduce and Retrain within the context of multivariate time series where time steps across the signals from the time series are removed.

Feature importance is trivial to validate when the ground truth or an expert opinion is available, this is known as faithfulness evaluation or fidelity evaluation^52^. However, ground truth is rarely available, which makes it otherwise difficult to validate without the use of other validation methods of feature importance. Existing validation methods of feature importance can be separated mainly into 2 categories, they are demonstrated in Figs. 1 and 2:

**Sensitivity Analysis**^25^, can be used to estimate feature importance. It is a form of ablation study^53^ that compares different subsets of features by measuring the difference in their performance achieved. A simple example will be when the subsets are derived in the manner of greedy combinations, where only combinations that yield a high performance are considered. Sensitivity analysis builds on the premise that performance of the model $\delta$ is expected to remain unchanged or minimally affected when using $D^*$, a subset which contains the important features. And the performance of the model $\delta$ is expected to drop significantly when using $D^`$, a subset which contains the least important features. Some examples of subsets that can be used to validate feature importance which follows the principle of ablation study, are Leave-One-Out (LOO) subsets and Singleton subsets. With LOO, this is when a single feature is left out of the input for the model $\delta$, where performance should experience a drastic drop if an important feature is dropped. With Singleton, this is when only a single feature is used as input for the model $\delta$, where performance should remain if an important feature is used. Together, LOO and Singleton subsets can be used to examine the importance of each feature within a dataset just as an extended ablation study would. Although logically sound, it is sometimes unfeasible to conduct Sensitivity Analysis with LOO and Singleton subsets due to computational cost and time from conducting high amount of subsets, especially if the duration of each subset is long. Sensitivity analysis can be guided by FS, where FS can serve as a more strategic way to select feature subsets for evaluation. A FS method is considered accurate or effective when the subset of features it selects can achieve a high performance when compared to others.

*Sensitivity Analysis with Perturbation methods*^54,55^ is a subcategory of methods which validates feature importance estimates by measuring the impact of perturbing features on the models prediction. Common approaches to perturb features are Gaussian noise, replacement with 0, etc. This is a popular approach as it doesn’t require retraining the model with each subset of features. An example of such is available in Fig. 1.

***Reduce and Retrain*** methods^26^, is another form of validation and ablation study which is more specific and systematic. The method is different such that the subsets of features are usually determined in a systematic manner. The number of features used in the subset for retraining is reduced in iterations in order to measure performance changes, specifically how the performance drops as the number of features reduces. In each iteration, the original model $\delta$ is retrained with less features to form subsequent models $\delta ^*$, which all have the same architecture but different model state due to retraining with less features. The rate of drop in performance from the retrained models $\delta ^*$ serves as an indicator regarding the quality of the FS method. If the rate of drop is drastic, it shows that the method is selecting important features for removal, whereas if the performance is retained over a long period of removal, it shows that the removed features were in fact redundant^54^.

In the context of multivariate time series, Reduce and Retrain is not always applicable. It can be applied to the channels of univariate time series, where less and less channels of univariate time series are used in subsequent retrain. This is shown in Fig. 2(a). However, it is not as straightforward when applied to the time steps. First, it is difficult to reduce specific time steps of one channel of univariate time series and retrain. Second, even if the issue is mitigated by removing time steps across all channels of univariate time series, the meaning of the results achieved is unclear. For example, if performance drops when a set of time steps was removed across all channels, which time steps from which channels were the cause? Also, did performance drop because important time steps were removed, or did performance drop because the order of the sequence is compromised and the data no longer make sense? An example of such is shown in Fig. 2(b).

## Related Work

Knowing what features are important and which are redundant is a major challenge, because this information is usually not known a priori^2,56^. Therefore we tend to use all features irrespective of the complexity^57^. NNs’ ability to process and learn direct mapping from the raw input to model prediction allows them to deal with such redundancy. This is attributed by their ability to conduct FS internally, as features are processed by the intricacies throughout its hidden units. The only issue being the limiting comprehension of the weighting of features with respect to the output of the model^58^. This is what the PIEE^22^ method previously aimed to achieve, to identify the features that are being selected internally and to estimate the importance of such features.

There are different ways to utilise the internal mechanism of NN to perform FS. There exists embedded methods which perform FS at the input level. They work by utilising a direct relationship between the input and some weights from the first hidden layer of a NN. This is reinforced by the hierarchy principal^23,24^ from statistic. When the NN is trained, the weights are regularised based on some constraint, such as Elastic Net regularisation, which then allows for a subset of the input features to be automatically selected. This utilises the internal training mechanism and regularisation to perform feature selection. Examples include, Deep Feature Selection^28^, LassoNet^43^, Sparse Centroid Encoder^59^, and variations of NNs with L1 regularisation that utilise the sparsity in features specifically to perform FS as the model trains. In such cases, features become “inactive”^44,60^ within the NN. Although this is desired in embedded methods of FS, the binary nature of the selection leads to an incomplete understanding of relative feature importance amongst the features. Additionally, since prior knowledge or expert opinion regarding the data is not always available, the binary nature of the selection not only cannot provide information regarding the relativity of importance amongst the features, it also makes it difficult to be used with verification approaches of feature importance due to the limited information available.

There also exists methods which utilise layering or the concatenation of NNs in order to perform FS. For example, in an existing work of Gui, et al.^61^, it was shown that a detachable Attention based mechanism^62^ is able to select features through an attention network between the input and a secondary fully connected network. This attention network correlates the input with the loss function between the output of the secondary network, and the supervised target to provide each feature a probability of being selected. In another work of Huang, et al.^29^, a neural network which performs threshold activation is embedded between the input and a secondary fully connected network to perform FS. Even in recent work of Khan, et al.^63^, NN of different architectures, including variations of CNN and Inception network, are used as feature extractor before feeding the extracted features, which are essentially selected features, towards a secondary fully connected network. The caveat of layering or concatenating NNs is the additional complexity that it introduces, especially concerning large networks with many parameters. In order for the layering of NNs to work as intended, this would first require computational cost and time to fine tune the parameters of the different NNs. This is not guaranteed to work and is subject to many different factors^64^. Upon the completion of fine tuning, if it is successful, it would require additional computation cost and time to run the layered or concatenated NNs.

Alternatively, feature importance can be assessed post-hoc, i.e., after the model has been trained. Feature attribution methods, such as DeepLIFT^27^, IntegratedGradients^46^, or Grad-CAM^65^ are methods that aim to understand a model through interpretability. This can be achieved by attributing an importance score to each input feature, in order to represent how much an input contributes towards a specific prediction of the model. These attribution maps can then be aggregated to reveal data properties that the model relied on for its predictions^66^. Moreover, feature attribution methods are the preferred type of explanation method in practice for understanding model behavior with the purpose of debugging and verifying models^67,68^.

The aim of the previously proposed method, PIEE^22^, was to identify feature importance without relying on prior knowledge or expert opinion. To allow for a more complete understanding by quantifying relative feature importance via the different analyses available from PIEE. PIEE could be embedded into NN that would capture the information necessary during the forward and backwards propagation of training. This incurs minimal computational changes and minimal architectural changes to the existing NN, which is specific to the use case. This would also not require fine tuning of parameters. The same cannot be said with complex layered or concatenated NNs, which incur additional computational cost and time along with the demand of fine tuning in order to work.

### Challenges with Time Series

The additional information of order within a time series adds nuance to interpreting features. Due to the nature of causal relationship, it is more likely for a period of time steps to share importance and for different periods to have different significance. Additionally, multivariate time series do not only have time steps as features, but each univariate time series is also a feature with different significance within the multivariate time series. As mentioned in Section Time Series Modelling, the traditional fully connected network is insufficient when it comes to modelling temporal dependency between the time steps of a time series. This is the main reason RNN architectures was introduced. With the use of different architectures, other NN approaches toward feature importance were conceived. For example, Class Activation Maps (CAM^69^), which were used to analyse the time steps utilised in classifying four classes of cardiac rhythms from univariate Electrocardiogram (ECG) data using Convolutional Neural Netowrks (CNN), a type of network that performs filtering with kernels. CAMs highlight the feature importance by projecting the weights of the output layer back onto the feature map of the last convolutional layer. Sood, et al.^70^ proposed a model-agnostic approach for estimating global feature importance in temporal models. Tonekaboni, et al.^71^ proposed a framework which can compute importance for a given observed time series in relation to temporal shift based on predictive distribution of a multivariate time series blackbox model over time.

However, embedded NN methods have not had such advances. This is because the difference in input and architecture makes it difficult to transfer embedded NN methods as they are toward time series. In order for embedded methods to work, some adaptations are required. For example, approaches such as DeepSHAP^19^, GradientExplainer^20^, and TreeExpaliner^21^ have shown to be capable of highlighting feature importance within an univariate time series using shapley values^17^. However they are not transferable to multivariate time series without adaptation, an example is the adapted KernelSHAP^72^ which adapts shapley values for RNNs.

On the other hand, the approach of layering or concatenating NNs is minimally affected by the change in input and architecture due to time series, as the stand-alone functionality of the layers or the NN supports such changes. An example will be the framework of multilevel Wavelet Decomposition Network (mWDN) suggested by Wang, et al^73^. It is a network of time series decomposition via high and low pass filters. The high frequency components are collected for intermediate use. The low frequency components are further recursively decomposed. mWDN has inspired the works of Residual Classification Flow network (RCF) and Multi-frequency Long Short Term Memory (mLSTM), both also from Wang, et al., which make use of the intermediate high frequency components for further processing to perform time series classification or forecasting. Both works make use of the results from intermediate models, classifiers from RCF and LSTMs from mLSTM, to arrive at a final model output. As demonstrated, the mWDN framework does not rely on a particular architecture in order to function. This makes it more versatile when it comes to the processing of either univariate time series or multivariate time series. Another example is the Wavelet-DTW Hybrid attEntion Network^74^ (WHEN), which has 2 components: wavelet attention enabled learning module and neuralised dynamic time warping. The wavelet attention enabled learning module uses bidirectional LSTM, and the neuralised dynamic time wrapping uses attention mechanism. The results of each are combined to produce a final model output. Both components make use of RNN and other advances with temporal processing in order to process either univariate time series or multivariate time series.

Despite the advantages that layering or concatenating NNs can bring, the basis of such frameworks is still hindered by the additional complexity that it introduces. In the case of time series, especially multivariate time series, the high dimensional input combined with the increased complexity of required NN architecture incur further computational cost and time. This makes an already fragile and difficult situation worse.

In terms of interpreting the outcomes from NNs purposed for time series regression or time series classification. The interpretability of mWDN was achieved by performing sensitivity analysis in each layer with respect to its intermediate classifier. This was concluded by establishing some estimates of feature importance, or having a domain expert find a suitable explanation for the importance assigned by the algorithm to certain features. The general principles of Sensitivity Analysis^25^ have been covered in Section Feature Selection and Feature Importance. The issue of Sensitivity Analysis is that it is impractical when subset selection is unguided. This makes it sometimes infeasible to apply Sensitivity Analysis if it is to be done with a high dimensional dataset due to the high number of possible subset combinations. This approach of establishing some estimate of feature importance to achieve interpretability is rather common. Various interpretability methods^75^ have been applied to recurrent network such as LSTMs, and fully convolutional networks such as ResNets, where feature estimators were evaluated via perturbation analysis^54^. This is when the values of estimated features of importance were changed in subsequent training, and the confidence of the original and new classifiers were compared. The same was also conducted with estimated features of insignificance for verification.

Another challenge with multivariate time series is that it is often complex to extract or analyse importance of features in data channels and time steps $c\times t$. This is due to the interdependent relationship between the channels *c* and the time steps *t*. Each channel contains time steps, however this is not the case the other way around. More specifically, it is possible to reduce the number of channels without significantly impacting the time steps, however it is not possible to reduce the number of time steps without significantly impacting the channels. Furthermore, when assessing importance of time steps, if blocks of time steps, also known as a period of time steps, exhibit high importance and the rest of the time series demonstrates the opposite. It stands to reason that a large majority of the time series contain irrelevant information and can be ignored when it comes to processing. Conversely, if importance is randomly distributed, it is not clear what part of the time series can be ignored. Whilst interpretability methods are plenty, these are some issues of multivariate time series that have not been explored thoroughly.

There exists a topological approach by Ferrá, et al.^14^ using persistent landscape which identifies relevant landscape levels and matches them to corresponding time steps of the time series. This gating layer described in Ferrá, et al. is similar to the pairwise structure used in PIEE^22^. This supports that it is possible to utilise such principle and architecture to identify importance within time series. Within this work, we showcase how PIEE is generalisable with RNN architectures such that its importance estimate framework is applicable towards univariate time series, as well as multivariate time series with minimal additional complexity and modification. This sets it apart from other embedded methods, which are constrained by the additional dimensionality of time series.

## Method

In this work, we seek to interpret features within time series using PIEE^22^ and compare its importance estimate with existing embedded FS NN methods and a xAI method. As mentioned in Section Feature Selection and Feature Importance, features within a time series can be solely time steps for a univariate series, or they can be both the time steps and the different univariate series that constitute a multivariate series. Amongst the features of time series, the following research questions were pursued:

**RQ1** Can existing embedded FS NN approaches and PIEE identify importance within univariate time series? Do they agree with the results of xAI?

**RQ2** Can existing adapted embedded FS NN approaches and the adapted PIEE identify importance (i.e time steps and data channels) within multivariate time series?

**RQ3** Does the adaptation towards existing embedded FS NN approaches and PIEE negatively affect performance?

### Pairwise Importance Estimate Extension (PIEE) with Time Series

We propose that it is possible to extend the existing PIEE^22^ method to work with time series. The main changes that the extension brings forth includes:

- Adaptation towards the pairwise layer in order for it to be applicable towards time series.
- Importance estimate of time steps within univariate time series
- Importance estimate of individual time series within multivariate time series.
- Importance estimate of time steps for each time series within the context of the multivariate time series.

**Fig. 3:**
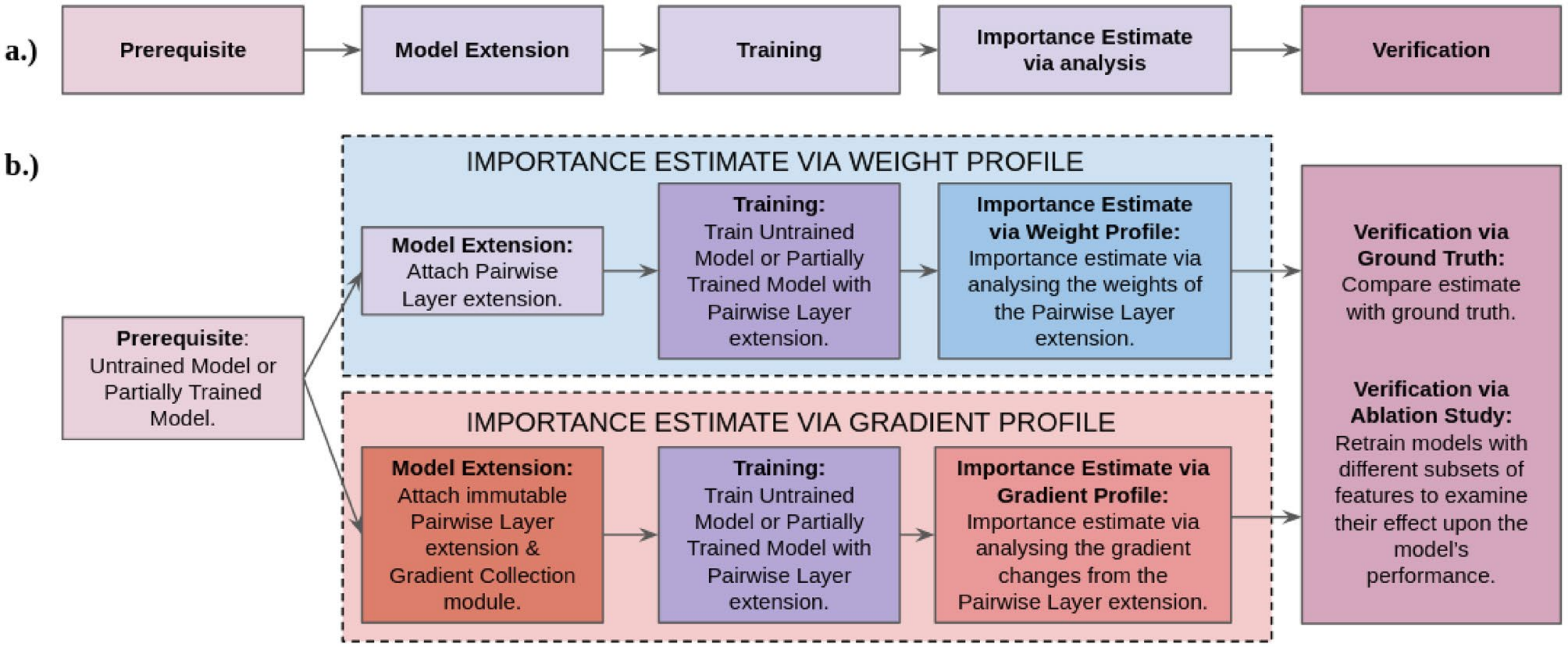
**(a)** Outline of the principles behind PIEE. (**b)** Outline of Importance Estimate via Weight Profile and Importance Estimate via Gradient Profile.

**Fig. 4:**
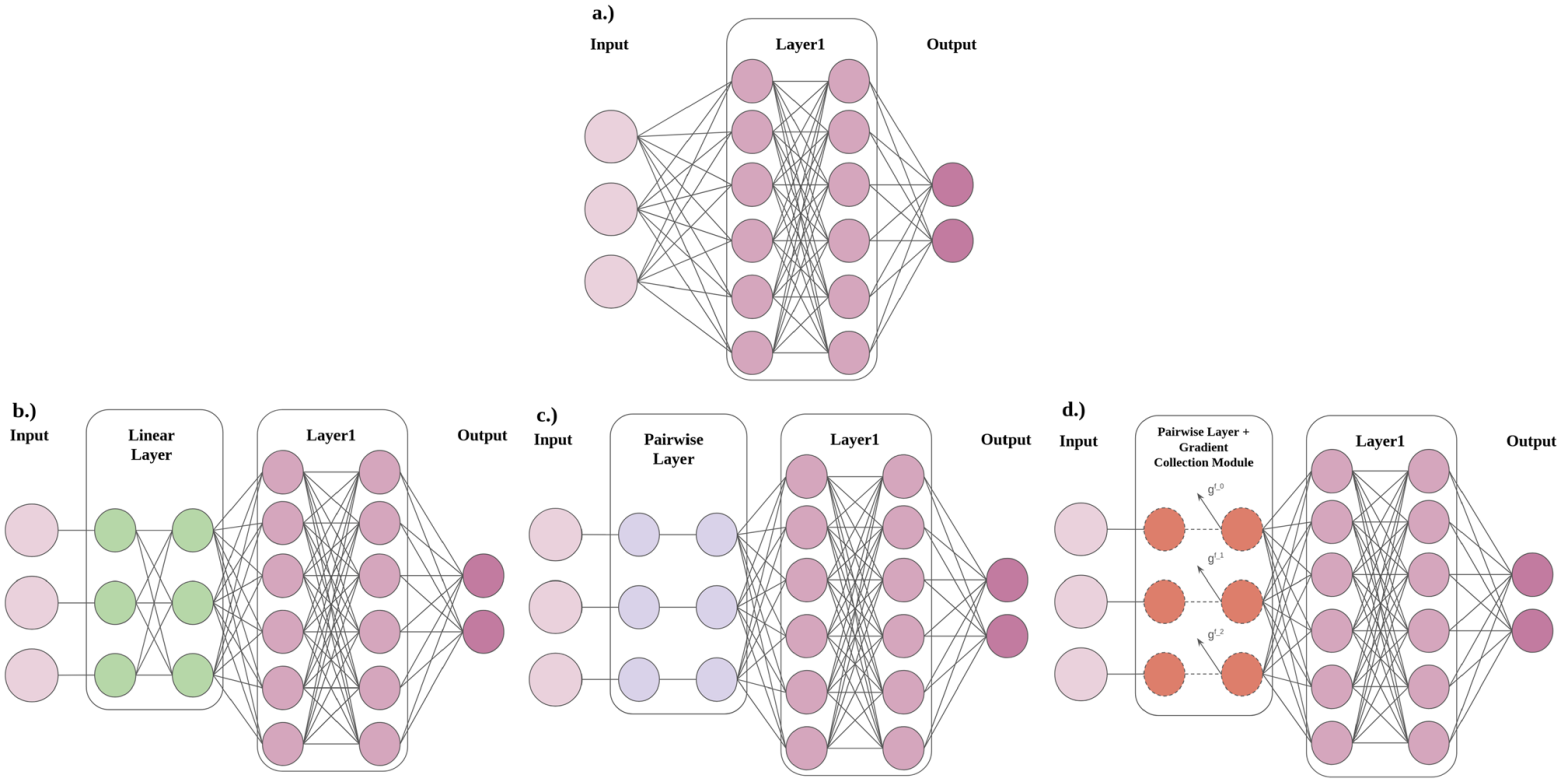
(**a**) A shallow NN. (**b**) A shallow NN with a linear layer between the input and the NN’s first layer. (**c**) A shallow NN with a pairwise layer between input and the NN’s first layer. (**d**) A shallow NN with an immutable pairwise layer between input and the NN’s first layer, where gradient changes for each respective feature are collected by the Gradient Collection Module.

We intended PIEE to act as a trainable extension that can be attached or detached from existing NN models, where the extension is generalisable towards all functioning NNs. An example of a functioning shallow NN is depicted in Fig. 4(a). Following the detachable novelty, this extension would not affect or would only have a minimal impact upon the integrity of the underlying functioning model. It would provide the means to observe the internal workings of the model, in order to compile information that reflects upon the relative importance of input features for the model. This is achievable via extracting information during backpropagation, which results in a fingerprint importance attribution based solely on the data used in training. This allows the extension to maintain the integrity of the underlying functioning model, which is different to existing methods that inadvertently obscure the information that the NN learn with additional complexity of NN layers or sparsity. This is useful in many cases as this introduces minimal complexity to the existing model, which is less likely to interfere with the parameters that were already fine tuned. The outline of the principles behind PIEE is as follows: The prerequisite is a functioning model that can perform its task, or a model that is partially trained for its task. Ideally, it is a model that can perform its task well, which is defined by having high classification accuracy and has not overfitted. This is followed by extending the model such that it becomes capable of producing importance estimates. In our case, this is the attachment of PIEE. This is then proceeded by training said model, where information for the estimate of feature importance is obtained. Finally, estimate of feature importance is calculated via analysis of the outcomes during training, and the estimates can be verified through different means such as ground truth or systematic methodology like Sensitivity Analysis^25^ or Reduce and Retrain^26^. This outline is illustrated in Fig. 3(a).

We proposed 2 modules of importance estimate: i) Importance Estimate via Pairwise Weight Profile and ii) Importance Estimate via Pairwise Gradient Profile. The 2 modules follow the same principles outlined in (Fig. 3(a)), however there are also fundamental differences in certain elements which affects the outcomes during training and their analysis, therefore their estimate of feature importance is different. The 2 modules of importance estimate that PIEE utilises to extend the underlying functioning NN are outlined in Fig. 3(b).

For the verification of estimated importance, we used the ground truth of importance when it is available. Otherwise, we used Reduce and Retrain^26^, a method based in the principals of ablation study, to examine the retention of performance as the subset of features estimated to be important decreases. We have included some of the core analysis from PIEE here for completeness.

**Required adaptation for multivariate Time Series – Prerequisite ** Since the number of units from the Pairwise Layer cannot be changed after initialisation, the input of the time series must have a fixed window size, or it must be processed into windows of the same length in order to fit the number of units declared upon the initialisation of the Pairwise Layer.

**Required adaptation for multivariate Time Series – Pairwise Layer** Due to the increased dimension within the input when it comes to multivariate time series, it is necessary to modify the Pairwise Layer which is responsible for Model Extension in Fig. 3. A pairwise layer is common and essential in existing works^28,43^ in order for the regularisation of the NN to feature select. Additionally, other works^29,61^ have demonstrated that information regarding feature importance with respect to the NN can be obtained between the input and the NN. This is also supported by the hierarchy principle^23,24^ from statistic.

In the case of feature datasets (unordered features datasets), which have instances *n* with *c* data channels as features. The pairwise layer functions as an element-wise multiplication, $\mathbf {X^{i} \odot C}$ where $\mathbf {X^{i}}$ is an input matrix with dimension $n \times c$, and *C* is a scalar variable with length *c*. The novelty here with the modification is that when it is applied to time series, specifically multivariate time series, the dimension of the input matrix increases. If it is an univariate time series, the pairwise layer functions similarly with the only difference being that the features in this case are time steps within the time series. However, when it is a multivariate time series, the dimension of the input matrix and the pairwise layer needs to match in order to perform element-wise multiplication. This requires the pairwise layer to adapt to the input matrix. For multivariate time series datasets with instances *n* of time steps *t* and data channels *c*. There are 2 variations available for the pairwise layer adaptation setup.

- The pairwise layer can perform **element-wise multiplication (EWM)** upon the data channels, $\mathbf {X^{i} \odot C}$ where $\mathbf {X^{i}}$ is an input matrix with dimension $n \times t \times c$ and *C* is a scalar variable with length *c*, which can also be represented as a vector of dimension $1 \times c$. Or element-wise multiplication upon the time steps, $\mathbf {X^{i} \odot T}$ where $\mathbf {X^{i}}$ is an input matrix with dimension $n \times t \times c$ and $\textbf{T}$ is a matrix with dimension $t \times 1$, which again can also be represented as a vector. Performing upon the data channels procures weights which represent the different time series in the multivariate dataset. This can be used to estimate the importance of each individual time series. This can also be performed upon the time steps to procure weights for the time steps across the different time series in the multivariate dataset. However this approach is unable to distinguish the importance of the time steps respective to each time series separately, therefore the estimate of importance for time steps via this approach is unrepresentative.
- In order to estimate the importance of time steps within a multivariate dataset with regards to each time series separately, the pairwise layer needs to adjust accordingly. It functions as $\mathbf {X^{i} \odot P}$ where $\mathbf {X^i}$ is an input matrix with dimension $n \times t \times c$ and *P* is the pairwise layer with a matrix of the same dimensions ($t \times c$). This is known as the Hadamard product, which returns a matrix of the same dimension with element wise multiplied entries. This approach treats each time step of each time series separately in the estimation of importance, which serves as a more complete estimate for the time steps within a multivariate time series. However, unlike element-wise multiplication upon the data channels, this approach is unable to provide a conclusive value of estimation regarding the importance of each time series (data channels). Further steps towards approximation is required, such as performing aggregation along the t axis to come to c values of importance estimate for each corresponding data channels. This alternate approach will be referred to as the **aggregated Hadamard product (AHP)**, and it will be used as a comparison to element-wise multiplication upon the data channels.

The difference between the adapted existing embedded FS NN and the adapted PIEE, is that PIEE, same as previously, does not take the final weights from regularisation^43,44^ nor the weights from additional NN mechanisms^29,61^ at face value. Instead, an analysis is performed on the gradient descent that led to the final weights of the pairwise layer. In order for a comparable analysis to be conducted between each feature, the weights of the pairwise layer are initialised as 1 s, the identity function, in order to serve as a reference point of relativity. For simplicity and continuity from the previous work, the pairwise layer and the analysis of it will be referred to as the Importance Estimate, IE. When IE is applied in the classification, it can be presented as $f(x)= \text {NN}(\text {IE}(x))$, which can be simplified to $f(x)= \text {NN}(x \odot P)$.

It is important to note that the pairwise layer is a type of linear layer, however it is not just any linear layer^76^. Therefore the replacement of a linear layer with the pairwise layer will not yield the same outcome. This is showcased between Fig. 4(b) and (c). The difference is in the forward propagation and the backwards propagation.

For the forward propagation, a linear layer performs a matrix multiplication with the input, where the weights of the layer is in the form of a matrix $c \times m$, where *m* is configurable depending on the parameter of the linear layer. During that multiplication, an element-wise multiplication cannot be guaranteed unless the matrix had a specific initialisation and setting that creates and maintains the diagonal matrix.

For the backwards propagation, when the change in gradient is applied to the weights during gradient descent, the proposed approach aims to analyse the gradient changes from the weights to estimate importance. This is achievable with pairwise weights, where the changes in the respective weight can be interpreted as a direct change for the corresponding feature. This is however not achievable with other kind of weight matrices, as all entries of the matrix will experience changes from the gradient descent which adds an extra layer of obfuscation in tracking the corresponding changes to each feature. Therefore whilst a pairwise layer is a type of linear layer, not all linear layer is a pairwise layer nor does it mean all linear layer will conform to the same minimal through the forward and backwards propagation, hence why it is necessary to use a pairwise layer in order to employ PIEE.

**Unchanged Importance Estimate via Weight Profiles** IE via Weight Profiling uses pairwise connected weights to observe the updates from gradient descent upon each input feature, in order to create their corresponding weight profile. A weight profile is formulated through the pairwise layer extension between the input and the first layer of the model. In order to measure via the extension, the weights of the pairwise layer extension all share the same initialisaion of 1 s in the beginning of training. This effectively means that the extension begins as the identity function, and gradually changes through the backpropagation process of training. This forces all the weights from the pairwise extension, where each correspond to an input feature, to share the same starting point in order to measure relative importance between the input features as the backpropagation process occurs. The weight profile for each input feature is thus formulated. The outline of such is available in Fig. 3(b), and the structure of the NN is demonstrated in Fig. 4(c). The introduction of *IE* in classification models does not alter the existing loss function, i.e cross entropy, CE, but creates another trainable variable. This makes the loss function as follows, $\min _{w,b, \text {IE}} \ \sum _{(x,y) \in D} \text {CE}(y, \text {NN}(\text {IE}(x)))$.

*Weight based analysis* uses the pairwise weights from the pairwise layer extension to form respective weight profiles which estimate importance for the corresponding feature. Within this method, there are different approaches to control the magnitude of the weights during training to create different weight profiles, these are listed below:

- **Weight-Naive** This is when no restriction is placed upon the weights during training, such that all gradient changes are propagated forward and backwards. For comparison with other approaches, the ranking of importance is based on the weights’ absolute magnitude, ranked in descending order. As this approach relies heavily on assumptions between the input features and the corresponding weights, it is more suitable as a baseline reference.
- **Weight-Constrained** This is when some restriction is in place for the weights during training, for example regularisation^28,43,44^. For comparison with other approaches, the ranking of importance is based on the constrained weights’ absolute magnitude, ranked in descending order.

The formulation of ranking for *Weight based analysis* is as follows: Let *F* be the number of features, where each feature has a score of importance. Let $s_x$ be the scores where $x \in F$ which is based on the absolute magnitude of the feature’s corresponding weight $|w_x|$. The ranking of feature *R* is defined as $(s_x)$ such that $s_i> s_j$ when $i,j \in F$ and $i<j$. Features can be selected based on their score’s ranking, where a higher score would mean higher significance, and vice versa.

**Unchanged Importance Estimate via Gradient Profiles** Similar to IE via Weight Profiles, IE via Gradient Profiles also uses pairwise connected weights to observe updates from gradient descent upon each input feature. However it achieves this differently by observing the updates from gradient descent without modifying the weights themselves via the addition of a Gradient Collection module within the Pairwise Importance Estimate Extension. This allows a gradient profile of each input features to be created from training. This is demonstrated in Fig. 4(d).

The reasoning behind IE via Gradient Profiles is to be able to observe the process of backpropagation without interfering with the integrity of the original model, and to observe the process in greater detail. A weight profile produces an importance estimate for a corresponding input feature by realising the gradient changes from the corresponding pairwise weight during the backpropagation of training. However, by doing so, it changes the original model as the forward propagation will be affected. Furthermore, a weight profile is only concerned with the pairwise weight at the end of training, which limits the amount of perceivable information that can be used for further analysis. The pairwise weights from the pairwise layer allows information of each input feature to be reflected in the backpropagation of training, and the Gradient Collection module offers the alternative of observing all information from training without modifying the integrity of the model. Similar to the process of formulating weight profiles, gradient profiles are also formulated through the pairwise layer extension between the input and the first layer of the model. In addition to the initialisation of 1 s for the pairwise weights, the pairwise layer extension is also intialised to be immutable. Again, this effectively means that the extension begins as the identity function in order to measure relative importance between the input features. Moreover, with the pairwise layer being immutable, its weights will not be updated by the gradient changes of the backpropagation process. This means that the pairwise layer extension stays as the identity function throughout training, $\min _{w,b} \ \sum _{(x,y) \in D} \text {CE}(y, \text {NN}(x))$.

*Gradient based analysis* focuses on the gradient changes from the epochs of training. The gradient changes of the pairwise layer’s weights are collected from every batch of every epoch by a Gradient Collection module, so that further analysis can be conducted with the gradient changes stored, which follows from statistical practices^77^. The formulation of ranking for **Grad** follows the same basis. Let *F* be the number of features where each feature has a score of importance, *E* be the number of epochs, and $g_e^f$ be the gradient changes of every batch from the corresponding epoch *e* for the feature *f*, where $f \in F$ and $e \in E$. The gradient profile $G^f$ can be defined as $( \gamma (g_1^f) \dots \gamma (g_E^f) )$ where $\gamma$ is a function applied to the gradient changes of each epoch. Several ways to analyse the significance of each feature were explored:

- **Grad-AUC** This approach utilises the sum of gradient changes of each weight from every batch of each individual epoch to form a gradient profile for each feature. Feature importance is estimated by the difference in the gradient profiles, which is measured by the Area under Curve (AUC). AUC^78^ takes the gradient changes throughout the epochs of training into account, which provides a more complete image of importance as the NN is trained rather than only capturing what works for the NN by the end of training. The importance of features can be ranked by the absolute magnitude of the AUC in descending order. This can be formulated as follows: let $s_x$ be the scores of importance where $x \in F$, which is based on $|AUC(G^x)|$ where *AUC* is the area under curve function and $G^x$ is the gradient profile with the summation function $\gamma =\sum$. The ranking of features *R* is defined by $(s_x)$ such that $s_i> s_j$ when $i,j \in F$ and $i<j$. Features can be selected based on their score’s ranking, where a higher score would mean higher significance, and vice versa.
- **Grad-ROC** This approach utilises the sum of gradient changes of each weight from every batch of each individual epoch to form a gradient profile for each feature. However, instead of directly using the AUC, the difference between the epochs are measured and analysed^79^. The AUC of such derivative is then used to measure the importance of features in the form of Rate of Change (ROC). Again, the importance of features can be ranked by the absolute magnitude of ROC in descending order. This can be formulated as follows: let $s_x$ be the scores of importance where $x \in F$, which is based on $|AUC(ROC(G^x))|$ where *AUC* is the area under curve function, $G^x$ is the gradient profile with the summation function $\gamma =\sum$, and *ROC* is the derivative of the gradient changes between the epochs such that $ROC(G^x) = ( \sum {g_1^x}, \sum {g_3^x}-\sum {g_2^x} \dots \sum {g_{F-1}^x}-\sum {g_{F-2}^x},\sum {g_F^x}-\sum {g_{F-1}^x} )$. The ranking of features *R* is defined by $(s_x)$ such that $s_i> s_j$ when $i,j \in F$ and $i<j$. Features can be selected based on their score’s ranking, where a higher score would mean higher significance, and vice versa.
- **Grad-STD** This approach utilises the gradient changes of each weight by calculating the standard deviation (STD) between the batches of each individual epoch to form a gradient profile for each feature. STD is able to provide an alternate form of analysis^80^, where it does not only consider significant gradient changes within each epoch, but it also considers the variance between the batches of each epoch. With the STD, the AUC is again used to measure the changes in magnitude of STD throughout training. Similarly, the importance of features can be ranked by the magnitude in descending order. This can be formulated as follows: let $s_x$ be the scores of importance where $x \in F$, which is based on $AUC(G^x)$ where *AUC* is the area under curve function and $G^x$ is the gradient profile with the standard deviation function $\gamma =\sigma$. The ranking of feature *R* is defined by $(s_x)$ such that $s_i> s_j$ when $i,j \in F$ and $i<j$. Features can be selected based on their score’s ranking, where a higher score would mean higher significance, and vice versa.

**Summary of Changes**

- In order for PIEE to function with time series, it now has an input requirement for the underlying functioning NN. This is essential towards the Model Extension step in Fig. 3 in order to be applicable with time series. The input towards the functioning NN needs to have a fixed window size for the adaptation towards the pairwise layer to be incorporated.
- Within a multivariate time series context, the adaptation towards the pairwise layer determines the type of analysis that can be utilised for the importance estimate of the time series.
  - Adapting the pairwise layer to be a vector allows for element-wise multiplication upon the individual time series. This estimates the relative importance of each time series present within the multivariate time series.
  - Adapting the pairwise layer to be a matrix allows for the Hadamard product to be calculated, which estimates the relative importance of the time steps between each individual time series within the multivariate time series. The importance estimate from the time steps can be further aggregated to form an overall importance estimate of each individual time series within the multivariate time series.
- The analysis of IE via Weight Profiles and IE via Gradient Profiles remains unchanged. The general framework of how importance estimate is calculated from the profiles remains unchanged. This allows PIEE to remain a generalisable method with minimal complexity for any functioning NN.

### Verification

The presence of a ground truth or existing domain knowledge makes the process of verification trivial, however this is not guaranteed. In the absence of any ground truth or existing knowledge specifying which input features are important to the objective function of a model, both Sensitivity Analysis^25^ and the Reduce and Retrain^26^ approach can be used to verify claims of importance. Their function and limitations have been covered in Section Feature Selection and Feature Importance. In order to verify the proposed adaptation and claims of PIEE being able to estimate feature importance within a time series context, we carried out a comparison study between a dataset with ground truth, a dataset with existing domain knowledge, and datasets that can only be verified by an ablation study.

**Fig. 5:**
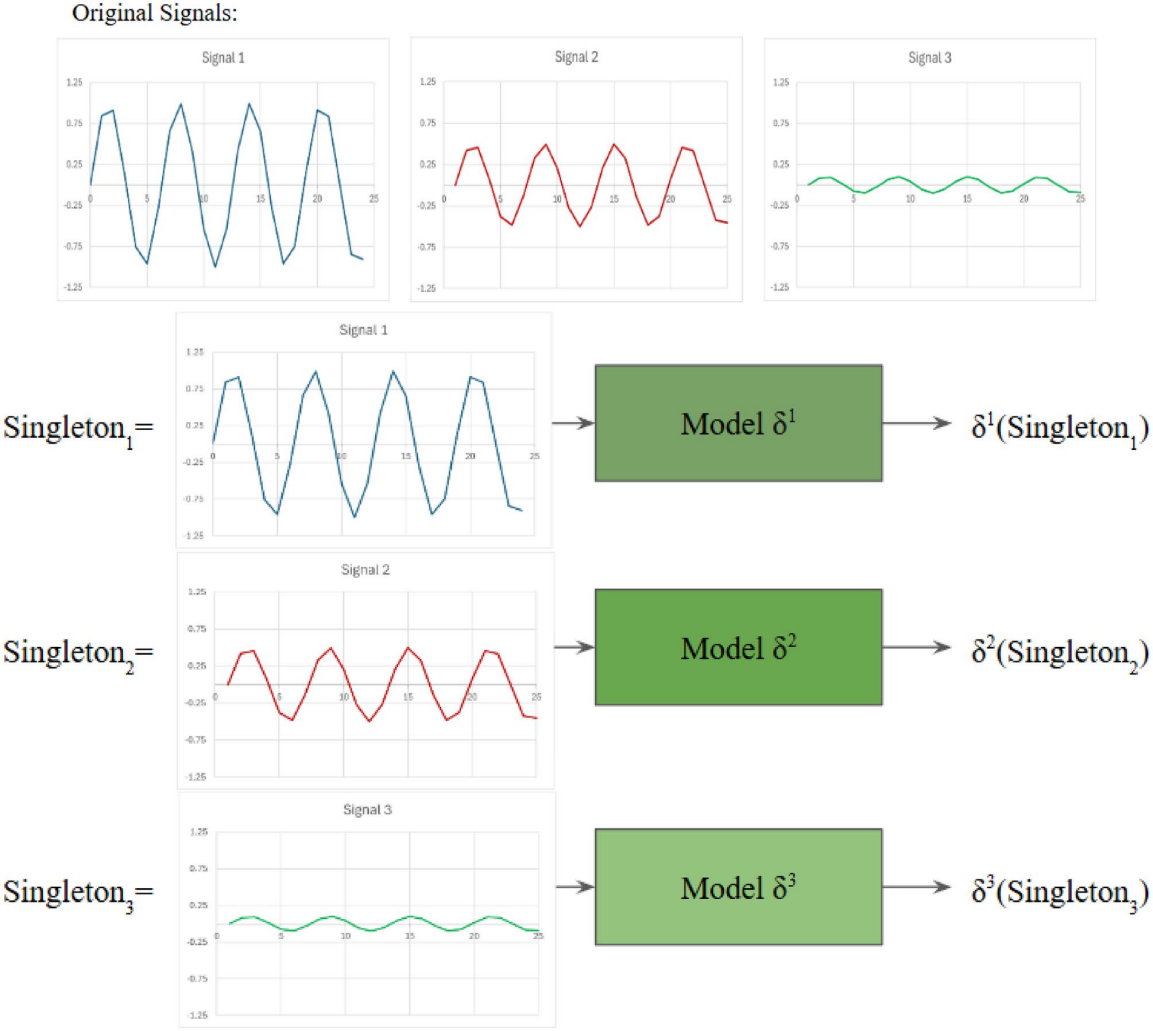
An example of Retrain with Singleton subsets within the context of multivariate time series, where only one signal is used as the input for retraining. This allows for a performance comparison, which informs the significance of the one signal used.

**Fig. 6:**
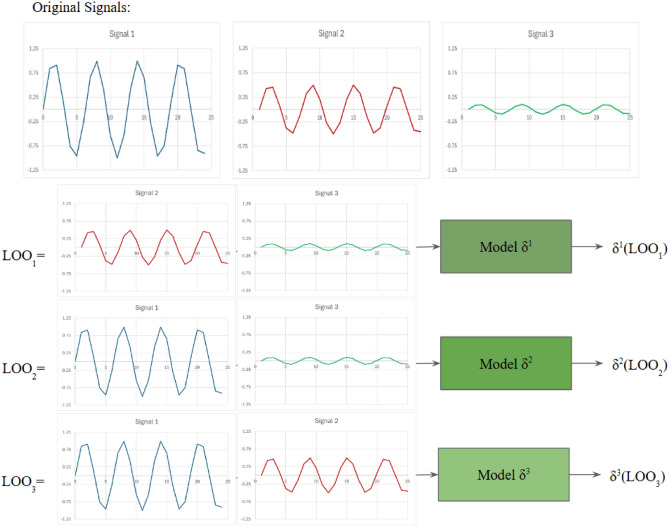
An example of Retrain with LOO subsets within the context of multivariate time series, where one signal is removed from the input signals, resulting in a LOO subset, which is then retrained for performance comparison. This informs the significance of the signal excluded from LOO subset.

We used a simulated dataset where the ground truth of feature importance for both univariate and multivariate time series context is available for verification. We also employed a real-world physiological dataset within the error decoding domain of neuroscience. This enables and provides a plethora of domain knowledge which can aid with the verification of significance within the physiological dataset. For the other multivariate time series datasets without ground truth or existing domain knowledge, we focused on the importance estimate of the data channels. Specifically, we used an ablation study^53^ that combines the principles of Sensitivity Analysis and Reduce and Retrain. This approach will be referred as Retrain with LOO and Singleton subsets. Which, as the name suggests, is based on systematically retraining with all the LOO and Singleton subsets, monitoring and using deviation in performance achieved by all the subsets to verify feature importance. This is illustrated in Figs. 5 and 6. This can be easily achieved by manually adjusting the input towards the model, and does not require changes to the model or the existing training process. However, even though it is a simple process, it can be very time consuming depending on the dataset. As each LOO and Singleton subset needs to be retrained, this results in a quadratic time complexity because it requires a duration of $2 \cdot (F \cdot E)$ where *F* is the number of signals and *E* is the number of epochs for the retraining. This makes it not viable with high dimensionality datasets or high volume of training.

In terms of verification of the pairwise EWM adaptation, as mentioned previously in Pairwise Importance Estimate Extension (PIEE) with Time Series, a time series $t \times c$ has 2 dimensions where *t* is the dimension of time steps and *c* is the dimension of channels (univariate time series). It is possible to perform element-wise multiplication upon the channels, or perform element-wise multiplication upon the time steps. However we have established that element-wise multiplication upon the time steps are unrepresentative and uninformative in the majority of cases, therefore we primarily focus on element-wise multiplication upon the channels for importance estimate within this work.

## Experiments

We carried out a comparison study with different experiments using different datasets. In the first set of experiments, outlined in Section E1: Simulated Time Series Datasets, we validated PIEE^22^ with multiple simulated univariate and multivariate time series datasets. Additionally, we compared the importance estimates with the established explanation method DeepLIFT^27^, as it has shown to produce faithful feature importance estimates for NN classifiers of time series^55^. In the remaining experiments, outlined in Section E2: AR EEG Error Decoding, Section E3: Occupancy Detection and Section E4: HHAR, we applied PIEE with 3 multivariate time series datasets from real-world applications.

In the experiments, we compared PIEE with different existing embedded FS NN approaches. The embedded approaches were adapted for multivariate time series processing when necessary. The respective estimates of importance for the channels from the different methods were compared, and the estimates of importance for the time steps from the different methods were compared when appropriate. Comparisons were made by evaluating with the ground truth or existing domain knowledge if they are available, or by verifying via an ablation study we referred to as Retrain with LOO and Singleton subsets, which combines the principles of Sensitivity Analysis^25^ and Reduce and Retrain^26^. This procedure is detailed in Section Verification.

### E1: Simulated Time Series Datasets

**Fig. 7:**
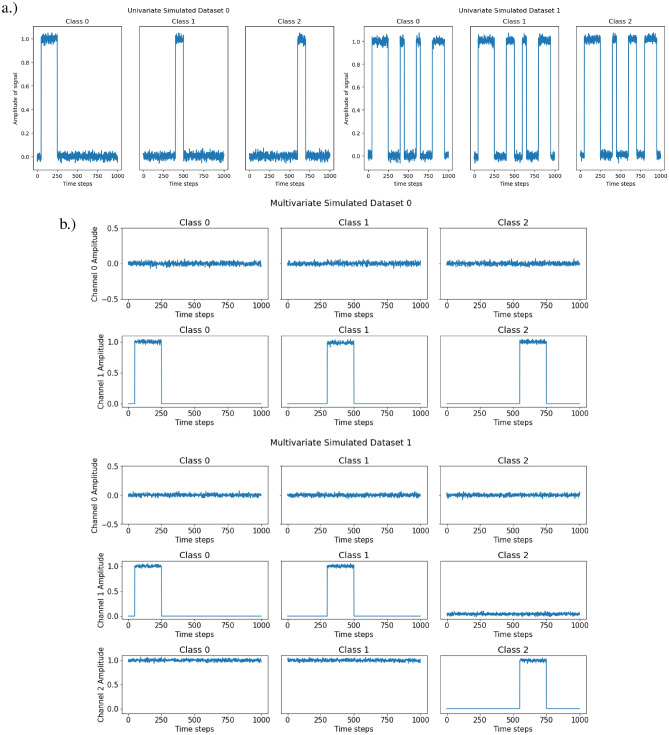
**Datasets** from E1: Simulated Time Series Datasets. (**a)** Simulated Univariate time series. (**b)** Simulated Multivariate time series.

Within this experiment, we simulated 2 univariate time series and 2 multivariate time series for classification. All of which have 3 classes with different characteristics. The 2 univariate time series with their 3 classes are illustrated in Fig. 7(a), and the 2 multivariate time series with their 3 classes are illustrated in Fig. 7(b).

**The goal of this experiment** is to prototype the pairwise layer adaptation necessary for time series, and evaluate the adaptation’s capability. More specifically, to identify importance within time steps of time series, as it is often difficult to verify such claims of importance in a time series. Simulated datasets were used in this case, such that the adapted PIEE^22^ approach and the adapted existing FS NN approaches could use the ground truth for verification.

In the simulated univariate time series of the experiment, there are 2 univariate time series with 3 classes. For univariate simulated dataset 0, we divided the classes based on different peak periods within the time series. For univariate simulated dataset 1, we divided the classes based on different signal formats, where a different pattern was used for each class. Both univariate simulated datasets are demonstrated in Fig. 7(a). The difference between the classes of the 2 simulated univariate time series was intended to test if the estimate of feature importance can identify the different patterns present in the simulated time series.

In the simulated multivariate time series of the experiment, there are also 2 multivariate time series with 3 classes. We divided the classes based on signal activity across the multiple univariate time series that constitutes the multivariate time series. For multivariate simulated dataset 0, channel 0 is a noise channel, this is to check if the estimate of feature importance will ignore the channel accordingly. This is demonstrated in Fig. 13(a). For multivariate simulated dataset 1, channel 0 serves once again as a noise channel with additional complexity for signal activity across multiple channels. This is demonstrated in Fig. 13(b). The 2 simulated multivariate time series are used as a proof of concept and ground truth for the adaptation, to see if it is able to identify patterns across multiple channels and ignore the noise channel.

The distribution of classes within each dataset was equal with 300 samples each for each class. Each instance has a time step of 1000 and $\mathscr {N}(0,0.02)$ was used to generate noise within the time series. We intended this dataset to not be very complex, as we wanted make sure that the proof of concept works as intended in a controlled environment.

**Training and Validation** The data was split 70% for training and 30% for validation. Stratified sampling of classes was used to ensure similar class distributions between training and validation sets. As the datasets used in this experiment are simulated, the ground truth of each dataset is readily available for verification.

**Model Architecture** A shallow neural network was used with a single linear layer and softmax activation. Adam was used as the optimiser with the default settings, and a learning rate of 0.001. The reason for the simple NN is to ensure that everything is as simple as possible to ensure minimum complexity in the testing of the adaptation prototype, such that should any errors arise, it will not be the fault of the network but the method.

### E2: AR EEG Error Decoding

In Wimmer, et al.’s work^81–83^, they conducted an interactive Augmented Reality (AR) study, where 20 participants manipulated a Rubik’s cube to study the neural responses to incongruent information in an AR scenario. The setup of the experiment included a tricolour Rubik’s cube (red, blue, white) and a camera to detect the nine colours that comprise its top surface. Participants sat at a table such that they could easily reach the cube and a keyboard. Users were visually instructed on how they should manipulate the Rubik’s cube. Afterwards, an augmentation was presented illustrating information related to the cube, which either matches the user’s expectations or not. A HoloLens 2^84^ was used to facilitate AR, and Unity^85^ was used to design the AR visualisations. It was also used to record gaze signals at 30 Hz. EEG signals were measured using a BrainAmp amplifier at 500 Hz with 32 electrodes positioning according to the international 10-10 system. EEG signals, gaze data, and markers from the experiments were synchronised as part of the pre-processing. Within their work, they conducted a 5 fold cross validation binary classification for each participant based on their EEG signals from the trials. They utilised 3 approaches for comparison: supervised linear discriminat analysis (sLDA^86^), support vector machine (SVM^87^) with a linear kernel, and **EEGNet**^88^. More details of their experimental setup, pre-processing, classifiers, and cross validation approach are available in their work^81–83^.

**The goal of this experiment** is to evaluate the adapted PIEE approach and the adapted existing FS NN approaches upon a binary classification of a multivariate time series within the EEG domain, to check if the estimate of feature importance from the adaptation is consistent in a practical real world dataset. In this experiment, there is no ground truth. However, the findings of Wimmer, et al^81,82^, as well as the plethora of neuroscience literature^89,90^, inform what electrode positions should be significant within the domain of EEG error decoding.

**Dataset** 20 volunteers participated in the study, however only 19 of the volunteers’ data were used due to erroneous behaviour from one of the volunteers. Each participant had 6 runs of 33 trials (23 congruent, 10 incongruent). The order of the trials was randomised. Each trial consists of 188 time points, its duration is between [−0.5, 1.0] seconds. Negative seconds represent the time before the occurrence of the stimulus, e.g congruent or incongruent signal response, and vice versa for positive seconds.

**Pre-processing** Only the EEG signals are of concern for this experiment, so gaze signals and other markers were ignored. The 32 EEG signals were scaled using the Standard Scaler from sklearn^91^ for each participant.

**Train and Validation** The cross validation approach used in the original work would yield too few instances for the training of neural network, therefore a user-fold cross validation approach was used. It is a K fold cross validation, where K is the number of users. Each fold was used once for validation, whilst the rest of the $k-1$ folds were used for training. The average of each fold was used for evaluating performance. In terms of verification, there is no ground truth. However, the findings of Wimmer, et al^81–83^, which are supported by other neuroscience literature^89,90^, have been able to inform the significant time periods of the signals, as well as the significant EEG channels from the EEG electrodes setup.

**Model Architecture**: We tried to replicate the EEGNet setup used in Wimmer, et al^81–83^ by utilising the EEGNet implementation from the *torcheeg* module from Pytorch^92^. EEGNet is a compact convolutional neural network built specifically for analysing EEG signals for neuroscience tasks, such as classification of event related potentials (ERPs). Within a standard EEGNet, there are 2 blocks of convolutional filters. This is followed by several parameters which define the structure of each block, such as the kernel size of each block, K; the filter number of each block, F; the number of depth-wise spatial filters, D; and the probability of dropout in its layers. In this work, the parameters we used for the EEGNet are: 32 electrodes; chunk size of 188 (number of data points in EEG chunk); 2 classes for binary classification; $K1, K2 = 32;$ $F1 = 8, F2 = 16;$ $D = 2; {Dropout\ rate} = 0.5$.

We later found through experimentation that the combined use of LSTM and EEGNet can yield better performance. This is evident from the performances achieved in Table 1. Further analysis of this is discussed within Section Verifying Importance via Existing Domain Knowledge.. The LSTM part from the LSTM-EEGNet architecture consists of a single LSTM layer with hidden units of 188, which is followed by a sigmoid activation function and a dropout rate of 0.2. A batch size of 64 was used for training the LSTM-EEGNet with an epoch of 300 along with the Adam optimiser with a learning rate of $5e-5$.

**Table 1:** F1 scores achieved by EEGNet and LSTM-EEGNet. Each row represents the F1 scores achieved by a participant. The columns, Weighted F1-score, present the weighted average F1 score of each participant. The average F1 scores achieved by all 19 participants are presented in the last row. The table shows that LSTM-EEGNet outperforms EEGNet in both the average Weighted F1-scores and the average incongruents’ F1 scores.

	EEGNet			LSTM-EEGNet		
	Weighted F1-score	Congruents’ F1	Incongruents’ F1	Weighted F1-score	Congruents’ F1	Incongruents’ F1
P1	0.685	0.865	0.506	0.673	0.852	0.494
P2	0.501	0.822	0.179	0.568	0.820	0.315
P3	0.500	0.810	0.191	0.558	0.808	0.308
P4	0.512	0.823	0.200	0.554	0.821	0.287
P5	0.468	0.822	0.115	0.557	0.827	0.287
P6	0.503	0.838	0.168	0.493	0.816	0.170
P7	0.574	0.805	0.343	0.591	0.805	0.378
P8	0.423	0.807	0.039	0.532	0.809	0.254
P9	0.571	0.849	0.294	0.648	0.854	0.442
P10	0.518	0.826	0.211	0.629	0.841	0.418
P11	0.532	0.835	0.230	0.531	0.823	0.241
P12	0.710	0.869	0.551	0.744	0.877	0.611
P13	0.581	0.832	0.331	0.615	0.833	0.396
P14	0.484	0.817	0.150	0.520	0.806	0.235
P15	0.511	0.829	0.194	0.527	0.809	0.244
P16	0.437	0.821	0.054	0.518	0.822	0.213
P17	0.613	0.834	0.393	0.608	0.830	0.386
P18	0.482	0.813	0.150	0.548	0.805	0.291
P19	0.591	0.845	0.337	0.678	0.856	0.500
**Average**	**0.536**	**0.830**	**0.243**	**0.584**	**0.827**	**0.340**

### E3: Occupancy Detection

**Fig. 8:**
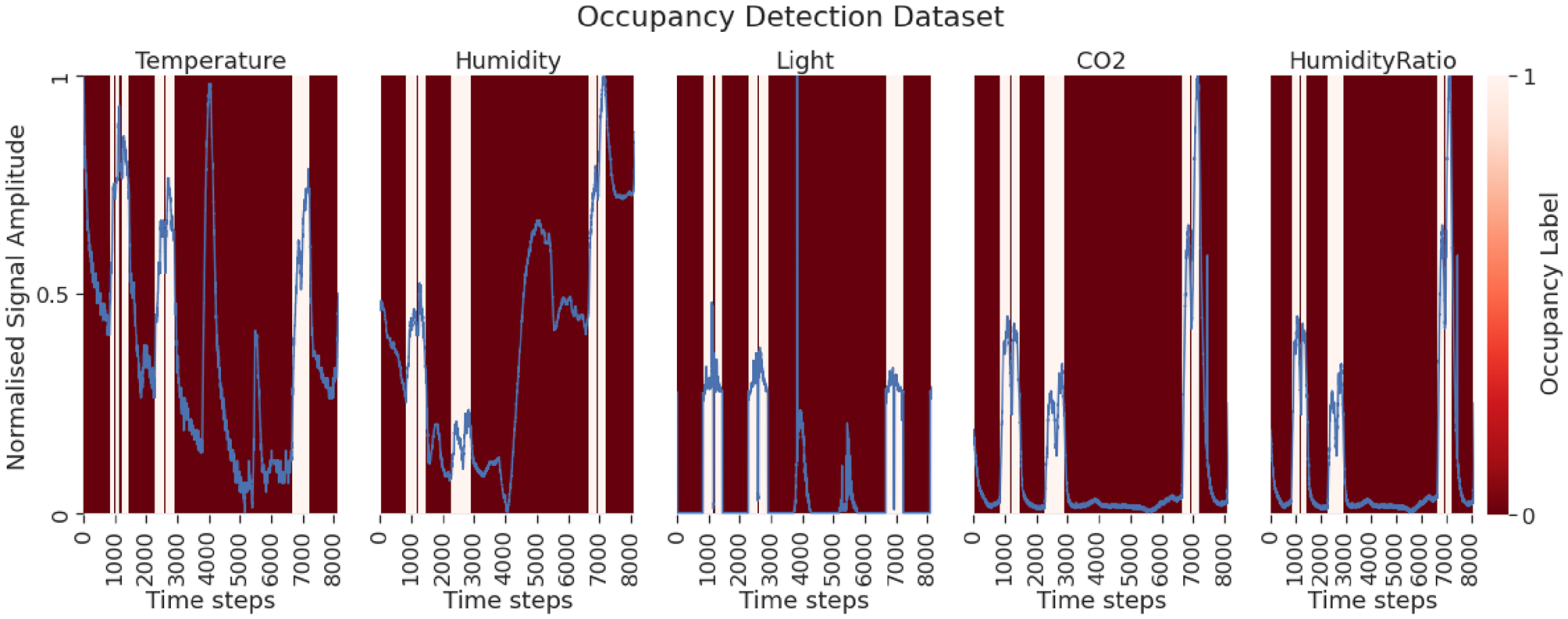
Occupancy Detection Dataset.

This is a dataset available from the UCI Machine Learning Repository^93^. It is a multivariate time series used for binary classification. The data consists of signals such as Temperature, Humidity, Light and $CO_2$, which correspond to the classification task of whether a room is occupied or not. The dataset is presented in Fig. 8 where the time series for the different signals are overlayed with a binary heatmap. Each time step of the multivariate time series is labelled. The white periods indicate “occupied” and the red periods indicate “unoccupied”. The multivariate time series is split into windows of size 64 with stride 1. The model takes windows of size 64 as input and produce a softmax vector output of dimensions, $64 \times 2$, given that it provides a binary classification of each individual time step.

**The goal of this experiment** is to evaluate the adapted PIEE^22^ approach and the adapted existing FS NN approaches with a real world multivariate time series that is a binary classification. In this case, even though the ground truth is not readily available, the features are informative regarding their own importance within the dataset, e.g occupancy is directly related to the level of $CO_2$. This information along with an ablation study using Retrain with LOO and Singleton subsets enables a comparison and verification of the approaches.

**Training and Validation** The data was split 70% for training and 30% for validation. Stratified sampling of classes was used to ensure similar class distribution between training and validation sets.

**Model Architecture** The model consists of an LSTM layer with 2 features in the hidden state, followed by a dropout layer set to 0.2 and the output layer. The output layer is a linear layer with a softmax activation. It was trained with the Adam optimiser with a learning rate of $1e-4$, and a batch size of 1 window sequence in order to perform a binary classification for each individual time step within the window. The reason for the simplicity of the configuration is to ensure that everything is as simple as possible. This is to ensure minimum complexity in the testing of the adaptation prototype, such that should any errors arise, it will not be the fault of the network but the method.

### E4: HHAR

This is also a dataset from the UCI Machine Learning Repsoitory^94^, it was used in the work of Stisen, et al.^95^ for the investigation of sensor heterogeneities for activity recognition. The dataset was devised to benchmark human activity recognition algorithms with respect to sensor heterogeneities. The dataset contains sensors, e.g accelerometer and gyroscope, of 8 smartphones,, and 4 smartwatches measuring 6 activities, e.g ’Biking’, ’Sitting’, ’Standing’, ’Walking’, etc., across 9 participants. For this experiment, we only used the data from the smartphones. The smartphones models include ’LG Nexus 4’, ’Samsung Galaxy S3’, ’Samsung Galaxy S+’, etc. The time series of the accelerometer and gyroscope were pre-processed and fused to provide a multivariate time series consisting of the axis data for both the accelerometer and the gyroscope. The features of the multivariate time series contain $accel_x$, $accel_y$, $accel_z$, $gyro_x$, $gyro_y$ and $gyro_z$.

**The goal of this experiment** is to evaluate the adapted PIEE^22^ approach and the adapted existing FS NN approaches with a real world multivariate time series that is a multi-class classification, to check if the estimate of feature importance from the adaptation is consistent. In this case, the ground truth is not available nor are the features informative as they are in E2: AR EEG Error Decoding. Therefore, an ablation study via Retrain with LOO and Singleton subsets is the only way to compare and verify feature importance.

**Preprocessing** The data was first cleaned by removing time steps with unidentified activity labels, and removing missing or NaN values. This was followed by scaling the sensor channels with Min-Max scaling. Afterwards, sequences of time steps were extracted from the accelerometer and the gyroscope. A sequence was defined by a series of data with less than 0.5 seconds difference between successive timestamps, and the series of data must have length greater than 1. The sequences were fused together based on their timestamps, which also tolerates a difference of up to 0.5 seconds. For the fused sequence, windows of events were extracted. An event was defined by a sequence which last more than 2 seconds. The average length of an event window was found to be roughly 262 seconds based on our procedure, and each time step is labelled with an activity class. Therefore the corresponding NN outputs a vector of $262 \times 6$, where 262 are the time steps and 6 because it is a multi-class classification. The code implementation of the preprocessing can be found in the Git repository available from the Data Availability Statement.

**Training and Validation** The data was cross validated by a variety of different approaches, which is detailed in the work of Stisen, et al.^95^. The main form of validation we use within this work is with the user fold cross validation approach. This is the same approach from E2: AR EEG Error Decoding, where the data is split into different folds based on users. A *K* fold cross validation, where *K* is the number of users. Each fold was used once for validation whilst the rest of the $k-1$ folds were used for training the model. The average of each fold was used for evaluating performance.

**Model Architecture** The model consists of an LSTM layer with hidden units of 64, followed by a dropout layer set to 0.1 and the output layer. The output layer is a linear layer with a softmax activation. It was trained with a RMSprop optimiser with a learning rate of $1e-4$, and a batch size of 1 window in order to perform a multi-class classification for each time step within the window. The reason for the simplicity of the configuration is to ensure that everything is as simple as possible to ensure minimum complexity in the testing of the adaptation prototype, such that should any errors arise, it will not be the fault of the network but the method.

### Methods for Comparison

For each dataset, comparisons were made between different feature importance methods.

- **Ablation Study via Retrain with LOO and Singleton subsets**, this is a method that focuses on the ablation of feature subsets and its effect upon the performance. LOO subsets systematically leave one feature out from the input of the NN, collect their respective performance as a result, and compare the performances with the other LOO subsets. The LOO subset with the worst performance indicates that the feature left out have affected the performance of the NN the most, therefore it must be of significance. Singleton subsets function in the opposite way. Singleton subsets systematically utilises only a singular feature from the original input of the NN, collect their respective performance as a result, and compare the performances with the other Singleton subsets. The Singleton subset with the best performance indicates that the feature used have affected the performance of the NN the most, therefore it must be of significance. The results from both Retrain with LOO subsets, and Retrain with Singleton subsets can provide a baseline regarding the importance of features. Specifically how much each feature contributes towards the performance of the model, which can be used for verification. This can be easily achieved by manually adjusting the input towards the model, and does not require changes to the model or the existing training process.
  - **Ablation study via removal of embedded architecture,** In addition to the ablation of features, further verification can be provided by the ablation of the adapted embedded approach from an embedded model. This results in the comparison of performance between the baseline model, the baseline model attached with the adapted PIEE approach, and the baseline models attached with respective adapted existing embedded FS approach. This informs whether the adapted methods have a significant impact on the performance of the baseline model.
- **Embedded FS based NN methods**, such as Deep Feature Selection^28^, **DF**, which uses regularisation on an Elastic Net and uses the weights from the Elastic Net to determine feature importance for FS. And NeuralFS^29^, **NFS**, which uses combined layering of nonlinear and linear function to produce weights that determine feature importance for FS. This approach of feature importance follows from *Weight based analysis* in Section Pairwise Importance Estimate Extension (PIEE) with Time Series. DF and NFS were chosen for comparison because they cover the fundamentals of embedded NN approaches. DF is based on the regularisation principle of embedded weights with respect to the input, and NFS is based on embedding layering of NNs. They are also the more stable variants from their respective approaches, which are less likely to be prone to issues such as fine tuning parameters and additional complication that were mentioned in 3 Related Work.
- **xAI**, the well established **DeepLIFT**^27^ was used as an additional qualitative comparison, when applicable, towards feature importance. DeepLIFT was selected since it has been shown to produce faithful feature importance estimates for time series classifiers^55^. DeepLIFT is a post-hoc local explanation method, i.e., it produces feature importance estimates for individual samples on an already trained model. Therefore, the mean feature importance computed across all samples of each class was used as comparison.
- **PIEE**^22^**’s Importance Estimate methods**, PIEE methods use a pairwise layer between the input and the model with different setup and training. From *Weight based analysis*, the importance of the features are estimated using **Weight-Naive**. This provides an additional baseline for comparison. From *Gradient based analysis*, the importance of the features are estimated using **Grad-AUC**, **Grad-ROC** and **Grad-STD**. The methods from the different analysis are described in Section Pairwise Importance Estimate Extension (PIEE) with Time Series.

Retrain with LOO and Singleton subsets, as well as Weight-Naive, will serve as the baseline measure for comparison. Both the LOO subsets and Singleton subsets serve as systematic analyses which measure importance in an opposite manner, therefore it is expected that they would share similar conclusive results. As previously mentioned in Section Verification, Retrain with LOO and Singleton subsets is a simple process, but it can be very time consuming when used upon high dimensionality datasets or when used with high volume of training. As each LOO and Singleton subset needs to be retrained, this results in a quadratic time complexity because it requires a duration of $2 \cdot (F \cdot E)$ where *F* is the number of features and *E* is the number of training epochs. The primary reason why this has been viable is because the datasets that we used in this work do not have an abundant amount of channels, which minimises the time it takes to carry out LOO subsets and Singleton subsets comparison.

Weight-Naive from PIEE is a simplistic approach that utilises the principles of *Weight based analysis* without additional constraints, hence why it serves as a baseline for comparison. Due to the similarity that all embedded NN based methods share, such as DF and NFS which follow from *Weight based analysis*, they are expected to perform similarly to Weight-Naive. Theoretically, the specificity of the additional constraints upon the pairwise weights in both DF and NFS should lead to a better performance than Weight-Naive. The same is also expected between the Grad-AUC, Grad-ROC and Grad-STD from PIEE, where the commonality of *Gradient based analysis* should result in similar performance between each other. As *Gradient based analysis* is a combination of *Weight based analysis* and statistical principles, it is expected that gradient based methods will perform better than the other embedded NN based methods.

It is important to note that the performance of the methods that utilise a NN model is dependent on the configuration and architecture of the NN used, as well as the amount of data available. The quality of feature selection from embedded FS based NN methods is influenced by the baseline performance that the deployed NN can provide. Therefore, the ablation study via removal of embedded architecture is expected to follow from the previous results^22^, where a minimum difference is expected from the introduction of the adapted pairwise layer extension for PIEE. However, it is uncertain how the adapted embedded FS NN methods, such as DF and NFS, will fare. It is also important to note that DeepLIFT was only applied to the simulated time series datasets due to incompatibility with the variant output setup of E3: Occupancy Detection and E4: HHAR.

### Performance Metric and Measure

Within this work, the primary focus is on the identification of feature importance within univariate and multivariate time series. This is best achieved when the ground truth or existing domain knowledge is available, where the identified features from the different approaches of Section Methods for Comparison can be verified directly. However, the ground truth or existing domain knowledge is not always available, especially regarding ground truths in real world scenarios. Therefore an alternative of using baselines for comparison will serve as an alternative form of confirmation. Specifically, the aforementioned Retrain with LOO and Singleton subsets, an ablation study which performs systematic retrain and analyse feature importance in opposite manners, together both LOO and Singleton subsets should inform conclusively regarding the significance of features for verification. The Weight-Naive method will serve as an additional baseline, as it is the basis of PIEE^22^ and should also inform the general capability of what different analysis of PIEE can achieve.

The secondary focus will be the comparison of performance between the existing embedded FS NN methods and PIEE. As adaptation was required in order to transfer the existing methods to be applicable to multivariate time series, the compatibility of the adaptation, which will be reflected in the performance achieved, will be examined and compared. We used F1-Score as the main performance metrics because it is the standard metric for classification result due to its combined use of precision and recall to take True and False Positives into account. In order to have a reliable measure of each method’s performance, 5 different train and validation splits were used for each dataset unless the split was done per user, i.e E2: AR EEG Error Decoding and E4: HHAR. Regardless of the difference in splitting, the average performance achieved by each method from the different splits of each dataset were used and compared.

Furthermore, when different methods have achieved very similar results, the Paired T Test^96,97^ was used in order to discern if there really exists a real significance between the results of the methods involved. The Paired T Test takes the distribution achieved by the methods, and compare them in a statistical manner to determine the t-value and the p-value. The t-value indicates if there is a difference between the distributions, and the p-value indicates the probability of achieving this difference. The p-value is used in conjunction with an alpha value $\alpha$, which is used as a threshold to determine whether the difference between the average of each method’s distribution can be considered significant. An initial alpha value $\alpha$ of 0.05 is used, which corresponds to a 95% confidence. Given that we perform multiple comparisons, we adjusted the alpha accordingly by using Bonferroni correction. Therefore, the null hypothesis is rejected when $\alpha < (0.05 / n)$ where *n* is the number of comparisons. We also included the Holm–Bonferroni^98^ method to correct the p-values to better account for the multiple comparisons.

The Paired T Test relies on the assumption that the pair of distributions have similar variance. This is defined by $\frac{1}{2}< \frac{\sigma _{1}}{\sigma _{2}} < 2$, where $\sigma _1, \sigma _2$ are the respective standard deviation from the pair of distributions to be compared with the test. When this is not the case, the Welch’s T Test^99^ is used instead, which allows for the pair of distributions to have different variance.

## Results

### Verifying Importance via Ground Truth

When it comes to verification of feature importance, the availability of ground truth makes the verification process trivial. However the ground truth is not always readily available, especially in real world scenarios.

Amongst the experiments, Section E1: Simulated Time Series Datasets contains simulated datasets with ground truth, which allows us to evaluate our adaptation’s proof of concept. It contains 2 simulated univariate datasets, presented in Fig. 7(a), and 2 simulated multivaraiate datasets, presented in Fig. 7(b), where we know the characteristics of each class within each dataset.

**Fig. 9:**
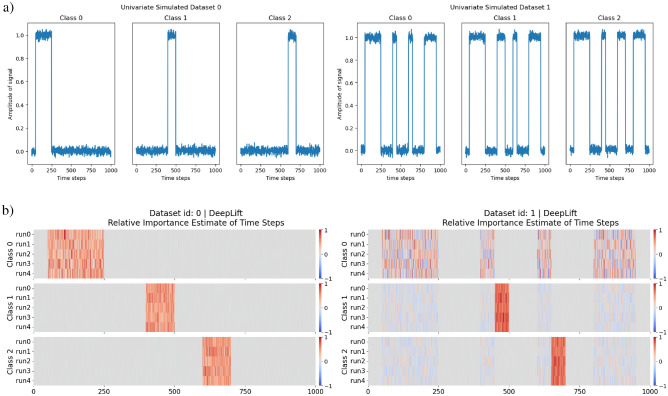
Simulated Univariate Datasets with DeepLIFT’s importance estimates: (**a)** The time series of **b**’s calculated importance estimate. (**b)** DeepLIFT’s importance estimate for each class of the time series across 5 runs with different splits. DeepLIFT captures succinctly the characteristic of each class.

**Fig. 10:**
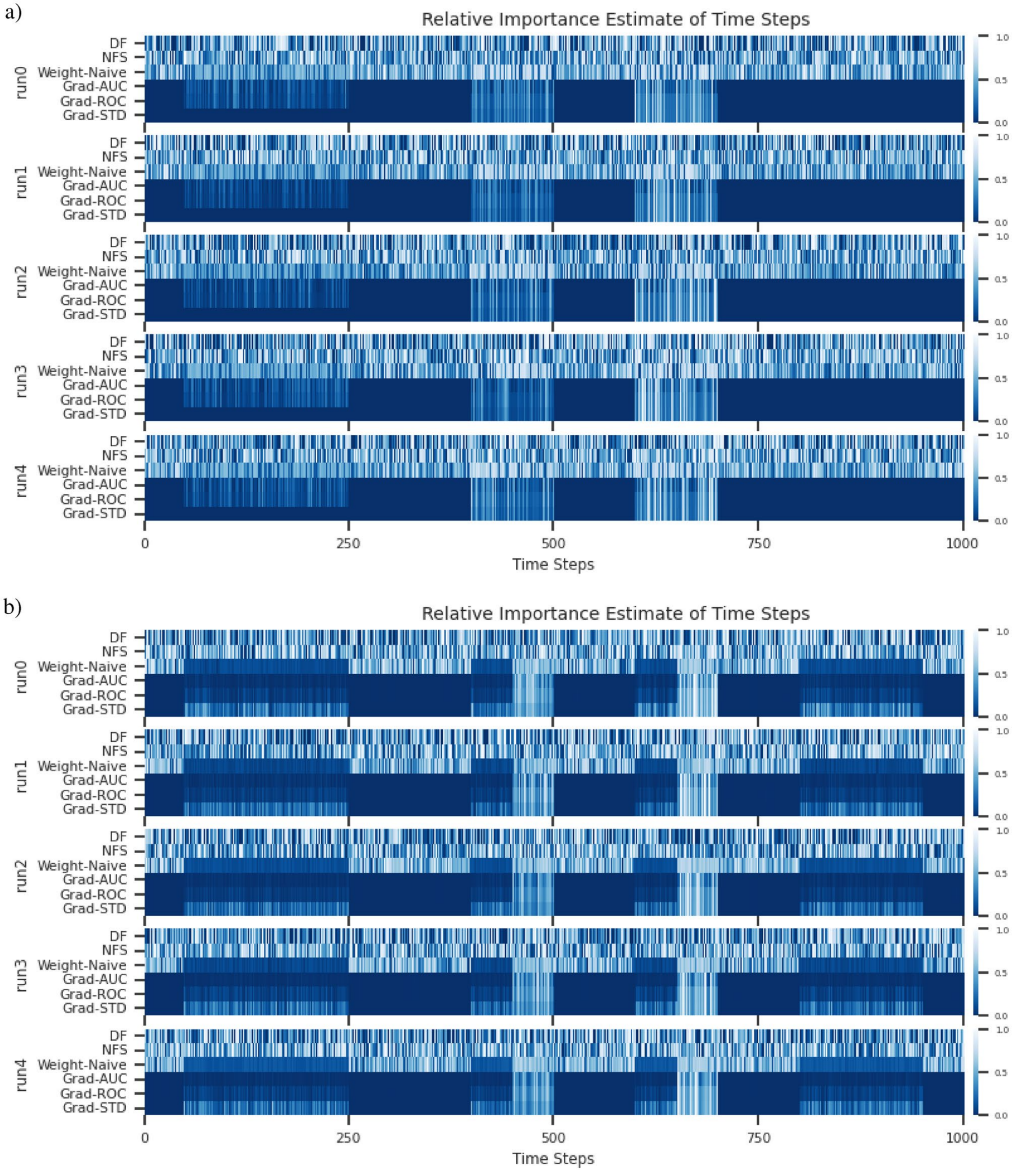
**(a) **Simulated Univariate Dataset 0 with existing embedded FS NN’s and PIEE’s importance estimate using AHP across 5 runs with different splits: PIEE’s Grad methods’ estimates are able to identify the key periods in differentiating the different classes, whereas others’ estimates are akin to noise. (**b) S**imulated Univariate Dataset 1 with existing embedded FS NN’s and PIEE’s importance estimate using AHP across 5 runs with different splits: PIEE’s Grad methods’ estimates are able to identify the key periods in differentiating the different classes. PIEE’s Weight-Naive method is able to capture some characteristic of the time series, although its estimate does not line up with the key periods.

For the simulated univariate datasets in Fig. 7(a), we can see that existing embedded FS NN methods, DF^28^ and NFS^29^, cannot produce conclusive estimates across the 5 different runs with different train and validation splits from Figs. 10(a) and (b). PIEE^22^’s Weight-Naive from *Weight based analysis*, on the other hand, is more conclusive across the 5 different runs, However, while class 1 and 2 can be detected, the feature importance estimate for class 0 is barely identifiable. Contrarily, the PIEE’s *Gradient based analysis *methods have shown in Figs. 10(a) and (b) to be conclusive across the 5 different runs and capture the main characteristics of each class. Additionally, For univariate dataset 0, the classes differ based on the different peak periods within the time series and *Gradient based analysis* methods in Fig. 10(a) have identified all the different peak periods whilst leaving the redundant periods untouched. For univariate dataset 1, the classes differ based on signal patterns, which are captured by *Gradient based analysis* methods in Fig. 10(b) where the main difference between the signal patterns are highlighted. Similar to previously, they have also left the redundant periods untouched. Additionally, we validated the model reliance on these features by comparing them with the class specific feature importances obtained with DeepLIFT^27^ in Fig. 9. Here, for univariate dataset 0, it is evident that the model relies on the correct features for each class. Also, for univariate dataset 1, it can be seen how the model attends to all high amplitude regions, while for class 1 and 2 it strongly focuses on each respective class’s discriminatory data properties.

For the simulated multivariate datasets in Fig. 13, we utilise the 2 different variations of the pairwise layer setup documented in Section Pairwise Importance Estimate Extension (PIEE) with Time Series: Element-Wise Multiplication upon the data channels, $\mathbf {X \odot C}$ where $\textbf{X}$ is an input with dimension $t \times c$ and $\textbf{C}$ is a vector variable with length *c*. Or the Hadamard Product, $\mathbf {X \odot P}$, where both $\textbf{X}$ and $\textbf{P}$ are of the same dimension $t \times c$. Across both multivariate datasets and both variations for the pairwise layer setup, it is clear that existing embedded FS NN methods, DF and NFS, with adaptation are unable to be conclusive across the different runs with different train and validation split, as well as not being able to capture the characteristics of each class for each dataset. Again, PIEE’s Weight-Naive from *Weight based analysis* on the other hand, is a lot more conclusive across the different runs although it is still lacking in capturing the characteristics of each class, as it is very prone to noise. The issue with noise becomes a lot more apparent when there are more channels present in the data. These findings are reflected by Figs. 13, 14 and 15. In contrast, PIEE’s *Gradient based analysis* methods are a lot more conclusive across the different runs with both variations of pairwise layer setup, and they are able to identify the characteristics of each class for both datasets.

**Fig. 11:**
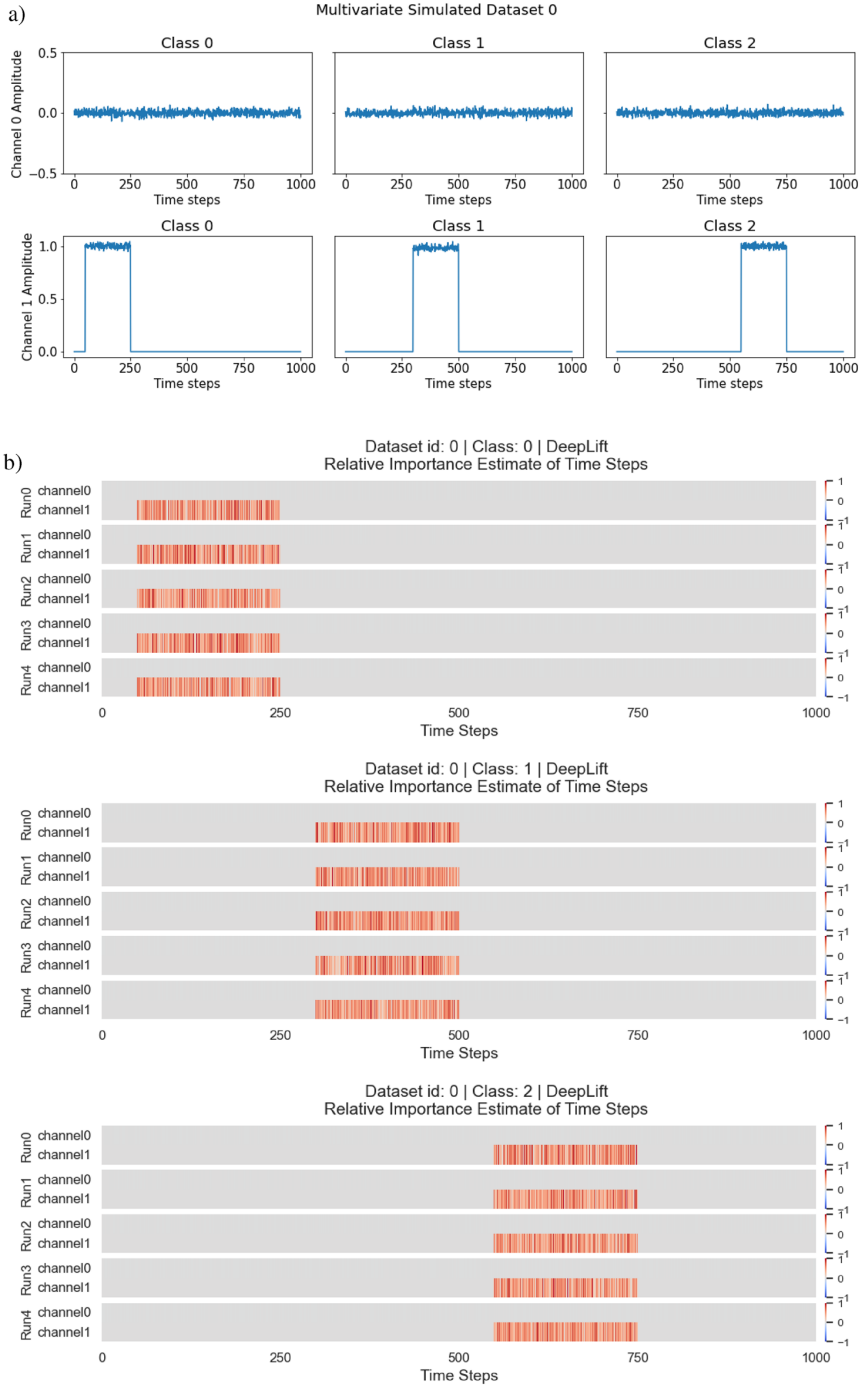
Multivariate Dataset 0 with DeepLIFT’s importance estimate: (**a)** The time series of **b**’s calculated importance estimate. (**b)** DeepLIFT’s importance estimate for each class of the time series across 5 runs with different splits. DeepLIFT captures succinctly each class’s characteristic for each channel.

**Fig. 12:**
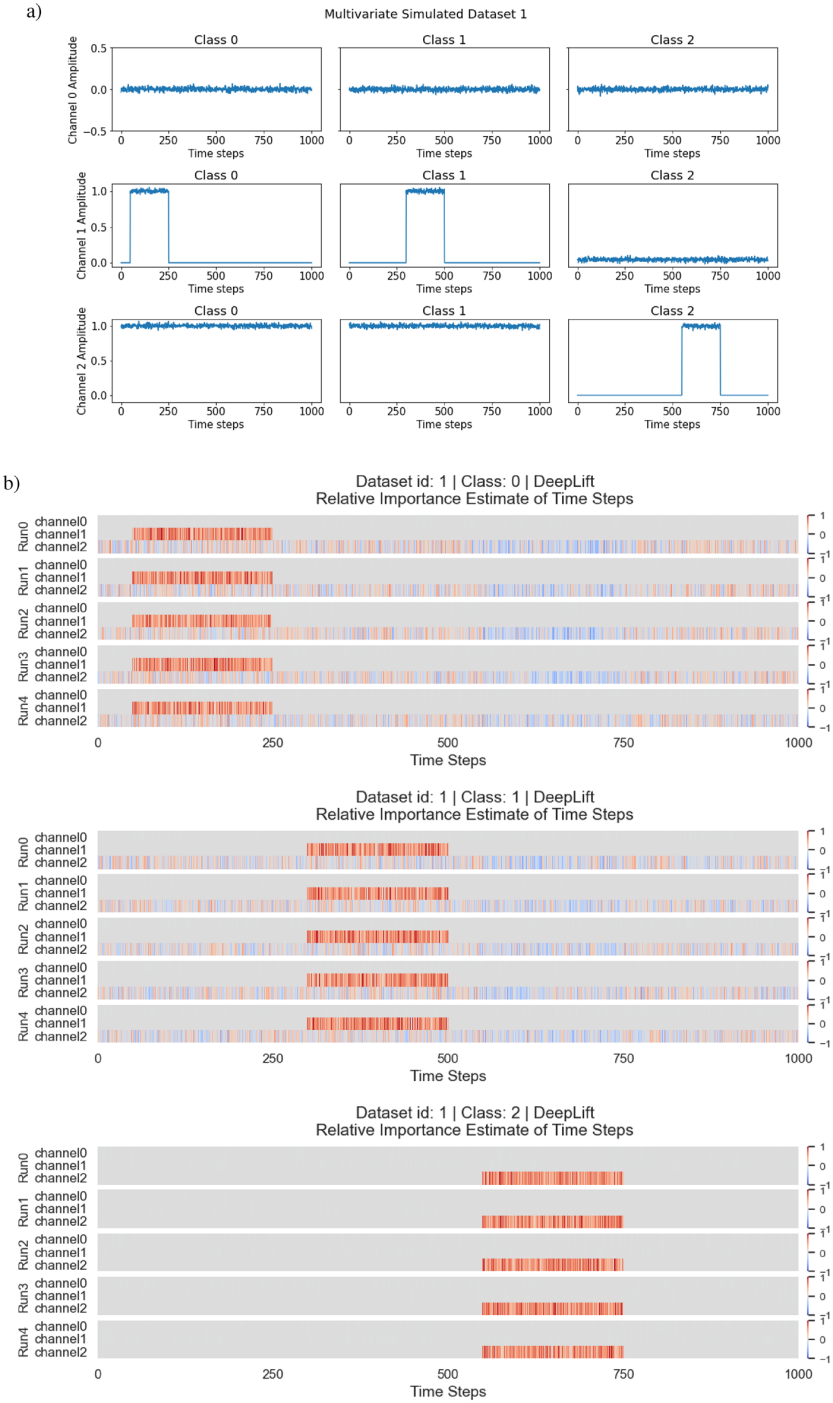
Multivariate Dataset 1 with DeepLIFT’s importance estimate: (**a)** The time series of **b**’s calculated importance estimate. (**b)** DeepLIFT’s importance estimate for each class of the time series across 5 runs with different splits. DeepLIFT captures succinctly each class’s characteristic for each channel.

**Fig. 13:**
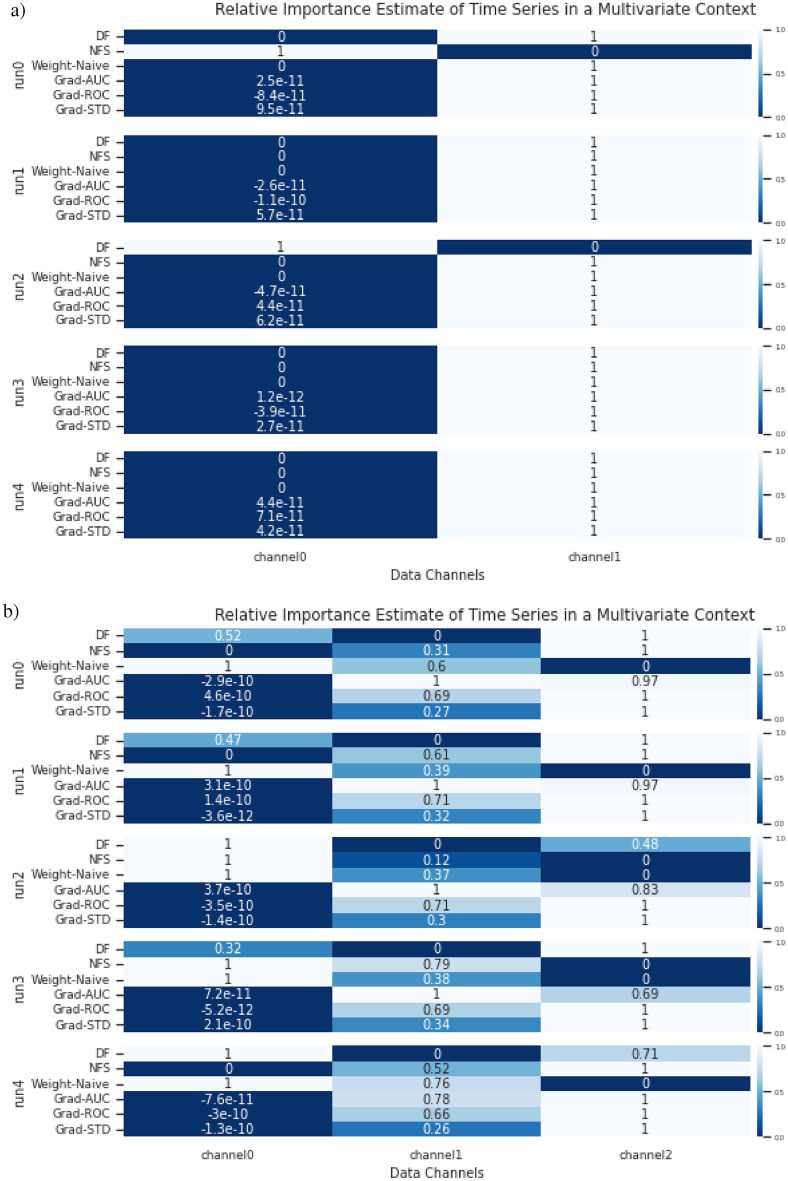
**(a) **Simulated Multivariate Dataset 0 with adapted existing embedded FS NN’s and PIEE’s importance estimate using EWM across 5 runs with different splits: PIEE’s Weight-Naive and Grad methods unanimously agree on their estimate, which is supported by the ground truth. DF and NFS are unable to do the same. (**b) **Multivariate Dataset 1 with adapted existing embedded FS NN’s and PIEE’s importance estimate using EWM across 5 runs with different splits: DF, NFS and PIEE’s Weight-Naive would produce high importance for the noise channel whilst PIEE’s Grad methods only produce high importance for the channels with relevant information for classification. This is supported by the ground truth of the simulation.

**Fig. 14:**
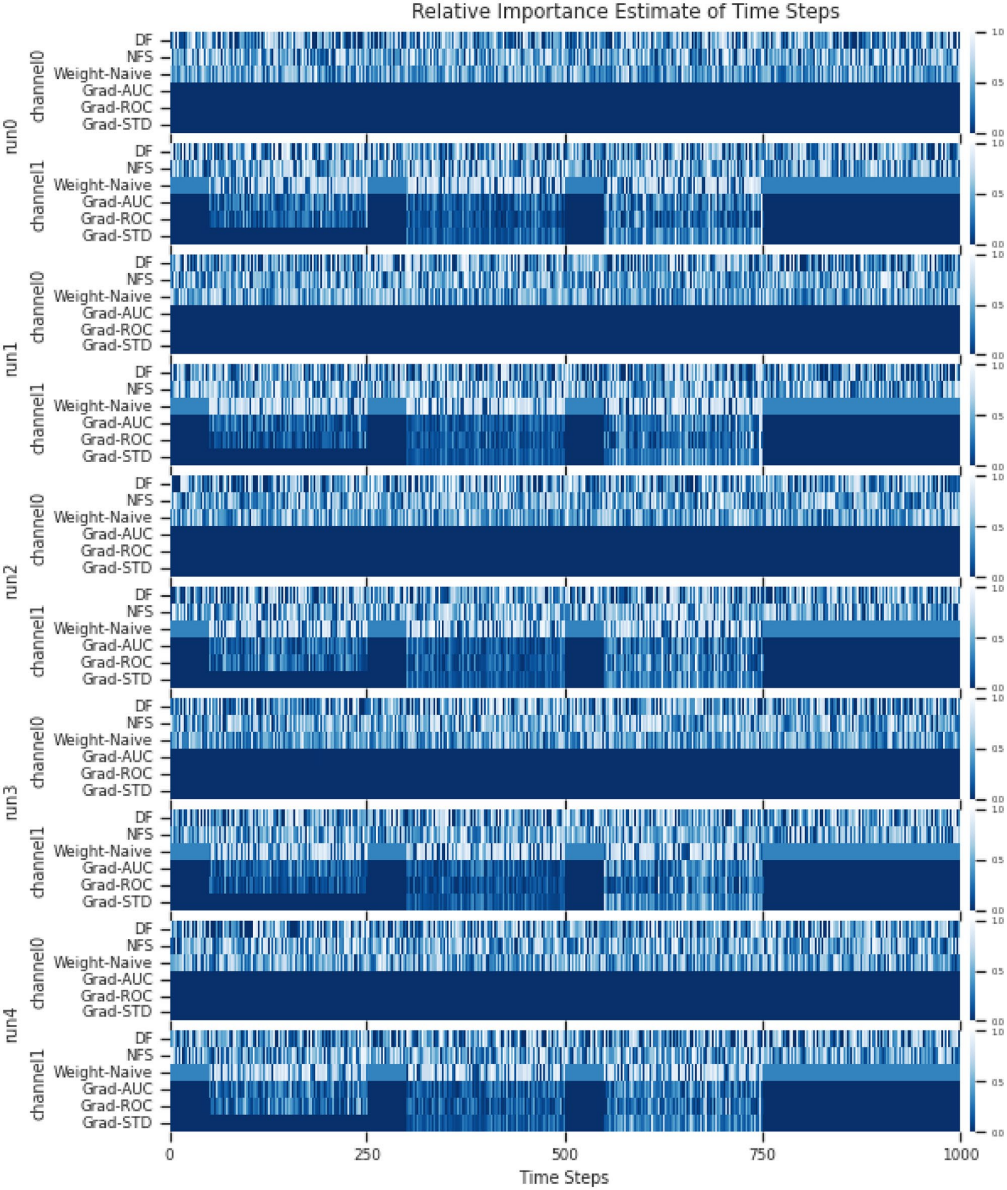
Simulated Multivariate Dataset 0 with adapted existing embedded NN’s and PIEE’s importance estimate using AHP approach across 5 runs with different splits: Grad methods agree on their estimate, which is supported by the ground truth of the stimulation.

For simulated multivariate dataset 0 and simulated multivariate dataset 1 via Element-Wise Multiplication upon the data channels, the *Gradient based analysis* methods are able to correctly ignore the noise channel, as well as being able to identify the channel(s) important for classification. This is evident from Fig. 13. For simulated multivariate dataset 0 and simulated multivariate dataset 1 via Hadamard Product, the *Gradient based analysis* methods are able to, again, correctly ignore the noise channels and identify the important time periods with respect to the time steps for classification. The result of simulated multivariate dataset 1 was especially insightful as the characteristic for classification is more complex where the importance of time periods can interchange based on the class. This is demonstrated in Fig. 14. Similarly to the univariate simulated data, we validated feature importance with DeepLIFT. Figure 11 depicts how all models learned to ignore the noise, and solely rely on the correct class specific data properties for multivariate dataset 0. Figure 12 also shows that the model learned to ignore the noise channel. Notably, the model correctly focuses on channel 1 when detecting class 0 and 1, whilst only focusing on channel 2 when detecting class 2. So, it was focusing on the right channel for each class.

**Fig. 15:**
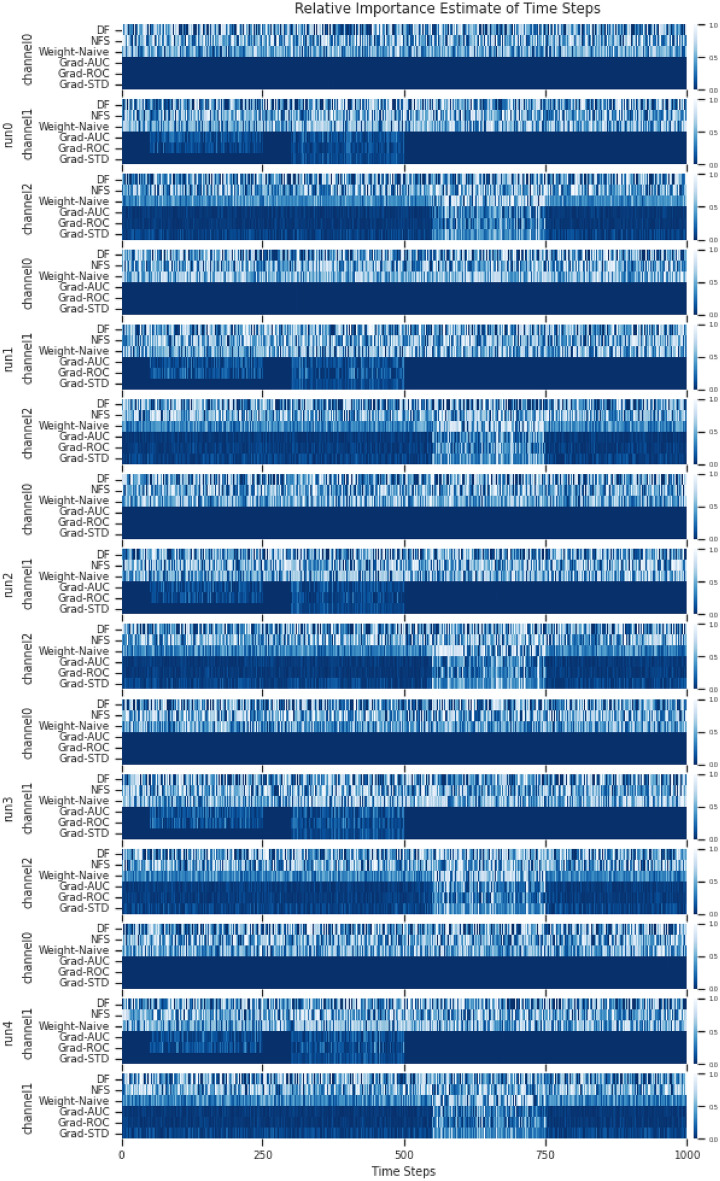
Multivariate Dataset 0 with adapted existing embedded NN’s and PIEE’s importance estimate using AHP approach across 5 runs with different splits: PIEE’s Grad methods agree on their estimate, and produce output supported by the ground truth.

### Verifying Importance via Existing Domain Knowledge

As ground truth is often limited in a real world scenario, existing domain knowledge can help. The experiment E2: AR EEG Error Decoding is an example of such cases, and it is extensively discussed within this section.

During experimentation, we have found that the stand-alone EEGNet^88^ setup from the works of Wimmer, et al.^81–83^ struggles to classify the incongruent class in particular. In further experimentations, we found that the additional use of LSTM helps to improve the classification of the incongruent class. The difference in classification F1 score is presented in Table 1. Statistical T Tests were conducted and the results show that there is a borderline significant difference between the average Weighted F1-scores of EEGNet and LSTM-EEGNet, which has t(5)=2.029; p-value=0.050 with $\alpha =0.05$. This significant difference is greater in magnitude in terms of p-value when considering the average incongruent F1-scores of EEGNet and LSTM-EEGNet, which has t(5)=2.338; p-value=0.025. These findings are important because PIEE^22^’s importance estimate is dependent on the working NN that it is attached to. However, it is important to note that even though LSTM-EEGNet can classify incongruent instances better than EEGNet, its F1 score is still lacking. This means that even though the importance estimates from PIEE applied to LSTM-EEGNet would be more informative than the importance estimates from PIEE applied to EEGNet, given the achievable F1 scores, even the importance estimates from LSTM-EEGNet are unlikely to be fully representative of the significance within the data.

In terms of importance estimate obtained from PIEE applied to LSTM-EEGNet, we investigated the 2 different PIEE variations of the pairwise layer setup documented in Section Pairwise Importance Estimate Extension (PIEE) with Time Series along with the other methods of comparison detailed in Section Methods for Comparison. In particular, we investigated significance in the data channels, i.e EEG channels in the context of E2: AR EEG Error Decoding, as well as significance in the time steps of the EEG signals.

One of the methods used as comparison was the DeepLIFT^27^. Given that DeepLIFT is a local post-hoc method, it was necessary to aggregate the explanations for the incongruent samples to arrive at an importance estimate measure. A participant-specific importance matrix of a given run can be generated by averaging feature importance scores across all incongruent explanations of a participant. Per-participant channel and time step importance can then be derived by averaging along the respective axes and normalising the results between [0,1]. Subsequently, the overall channel and time step importance can be calculated by averaging these per-participant values followed by [0,1] normalisation across all 5 runs.

**Fig. 16:**
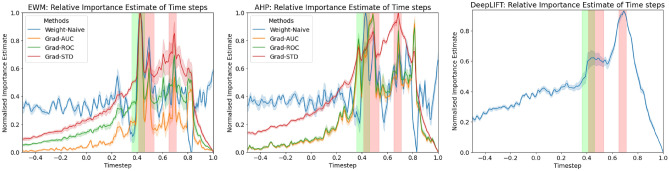
AR EEG dataset with adapted PIEE’s importance estimate of time steps using EWM (left), AHP (middle) and DeepLIFT (right) across 5 runs of user-fold split: red highlights periods with significant difference, green highlights event related theta peak. The graphs demonstrates that PIEE methods, as well as DeepLIFT peaked during the significant periods (red and lime).

Regarding significance within the time steps, this has already been established within Wimmer, et al.^81–83^ where significant differences have been found in the (red) segments of [0.412, 0.532]*s*, [0.652, 0.708]*s*, and event related theta (4-8Hz) synchronisation has been found to peak between the (lime) period of [0.360, 0.460]*s*. In Fig. 16, the time steps importance estimates of Weight-Naive from PIEE’s *Weight based analysis*, as well as Grad-AUC, Grad-ROC, and Grad-STD from PIEE’s *Gradient based analysis*, are illustrated together. The 2 adaptations: EWM and AHP, are shown in different graphs for comparison. The methods of DF and NFS are not included within the figure because their importance estimate output were both nonsensical with noise for both adaptations. Their importance estimates can be found in Figure S1 of the Appendix instead. From Fig. 16, it is evident that both adaptations for Weight-Naive of *Weight based analysis*, as well as Grad-AUC, Grad-ROC and Grad-STD of *Gradient based analysis*, have strong responses in their importance estimate during the important time periods that have already been established by Wimmer, et al. It is especially apparent that Grad-AUC’s, Grad-ROC’s and Grad-STD’s importance estimates all had stronger responses than usual between the time period [0.4, 0.8]. This reinforces that the adaptations of PIEE’s *Weight based analysis* and *Gradient based analysis* can identify significance within the time steps of multivariate time series. Furthermore, the time step importance estimates of Grad-AUC, Grad-ROC, and Grad-STD are also in agreement with DeepLIFT’s according to Fig. 16. Where the highest responses of DeepLIFT could be observed between the time periods, [0.412, 0.532]*s* and [0.652, 0.708]*s*.

**Fig. 17:**
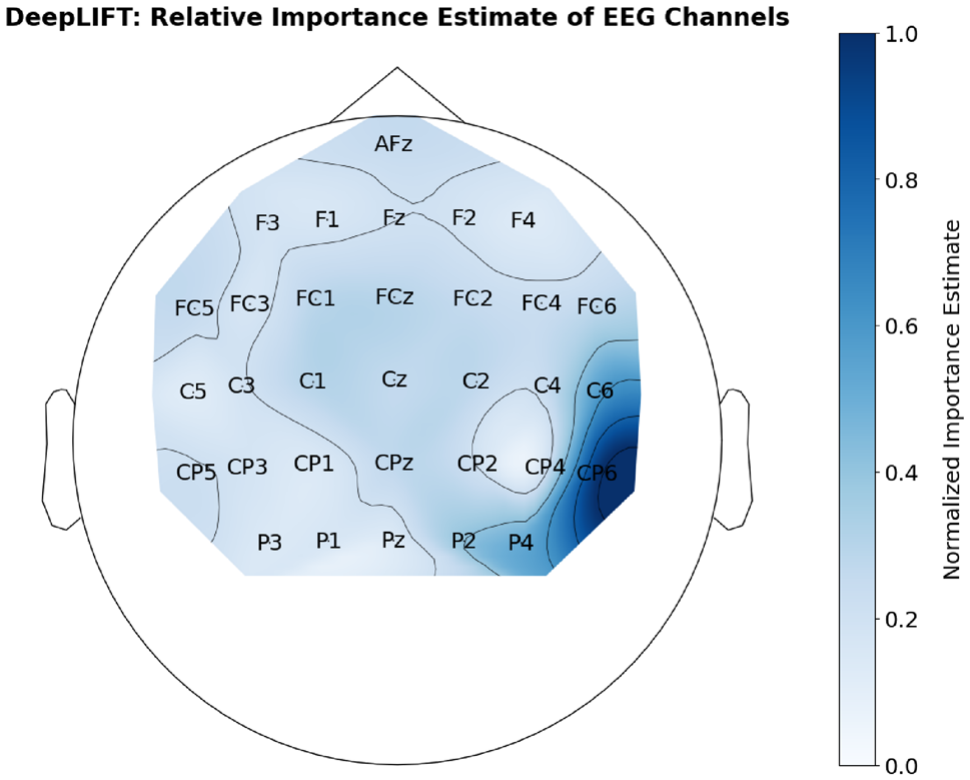
AR EEG dataset with DeepLIFT importance estimate of EEG channels across 5 runs of user-fold split: the dark blue areas represent higher importance estimate than lighter blue areas, as informed by the colour bar. The graphs show that DeepLIFT favours the right centro-parietal similar to the *Gradient based analysis* methods in Fig. 18.

**Fig. 18:**
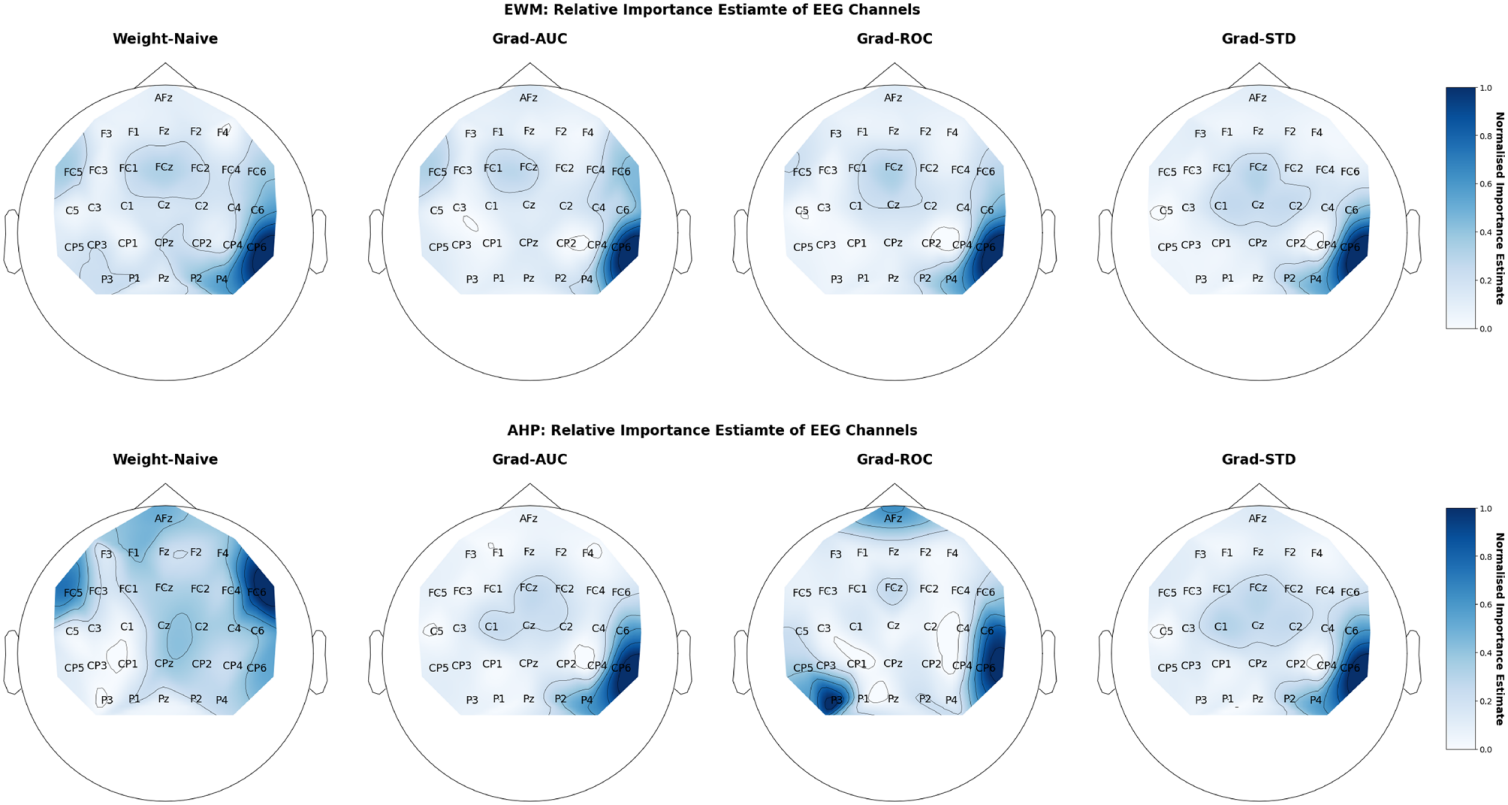
AR EEG dataset with adapted PIEE’s importance estimate of EEG Channels using EWM (top) and AHP (bottom) across 5 runs of user-fold split: the dark blue areas represent higher importance estimate than lighter blue areas, as informed by the colour bars. The graphs show that the *Gradient based analysis* methods tend to favour the right centro-parietal regions across both adaptations, whereas Weight-Naive of *Weight based analysis* is inconclusive across both adaptations.

Regarding significance within the EEG channels, this has also been established within Wimmer, et al.^81–83^ and existing literatures^89,90^ that centro-parietal areas (CP channels) are more responsive when it comes to error decoding tasks. The specific significant differences are captured by Fig. 2 from Wimmer, et al.^83^. Again, in Fig. 18, the EEG channels importance estimates of Weight-Naive from PIEE’s *Weight based analysis*, as well as Grad-AUC, Grad-ROC, and Grad-STD from PIEE’s *Gradient based analysis* are illustrated together. The 2 adaptations: EWM and AHP, are shown in different graphs for comparison. Similarly to previously, the methods of DF and NFS are not included within the figure because their importance estimate output were both as nonsensically noisy as their time steps importance estimate counterpart. Again, they can be found in Figure S2 of the Appendix instead.

Based on the findings and Fig. 2 of Wimmer, et al.^83^, the consistently significant EEG channels are located in the centre and right side of the centreo (channels Cz, C4, C6), centro-parietal (channels CPz, CP2, CP4, CP6) and the parietal areas (channels Pz, P2, P4). For the EWM adaptation, Weight-Naive from the *Weight based analysis*, as well as Grad-AUC, Grad-ROC, and Grad-STD from the *Gradient based analysis*, have estimated CP6 and its surrounding area (i.e C4, C6, P2, and P4) to be important. ROC and STD have additionally identified Cz and C2 to have some significance. Weight-Naive had an unique response to CPz having some significance. These findings are all inline with what was established in Wimmer, et al.^83^. Regarding the AHP adaptation, Grad-AUC’s and Grad-STD’s from *Gradient based analysis* are similar to its EMW adaptation counterpart, however Grad-ROC’s from *Gradient based analysis* and Weight-Naive from *Weight based analysis* are noticeably different. In the case of Weight-Naive, its importance estimates have identified importance to be in the frontal centreo (FC channels) and frontal (F channels) areas, which are areas that had some responses but they are not the areas where significant difference was consistently found from Wimmer, et al.^83^. And in the case of ROC, its importance estimates still mainly focused on CP6 and its surrounding areas (CP4, C4, C6), however its importance estimates also indicated importance in P3 and slight importance in AFz. P3 is still amongst the pareto areas which is important according to Wimmer, et al.^83^ but again it is not a consistently significant channel. The same applies to AFz as well, only that AFz is even more infrequently significant. When compared to DeepLIFT, the representation of EEG channel importance estimate demonstrated in Fig. 17 is very similar to Fig. 18, which consists of PIEE’s Weight-Naive, Grad-AUC, Grad-ROC, and Grad-STD representations.

It is important to note that differences between the significance findings from Wimmer, et al.^83^ and PIEE’s importance estimate could be attributed by a number of factors. Firstly, there exist differences in the setup between the work of Wimmer, et al. and our experiment. For example, event-related potentials (ERPs) were used to identify significance in Wimmer, et al. as opposed to the utilisation of raw EEG signals within this work. The 5-fold cross validation split used in Wimmer, et al. is also fundamentally different to the user fold split we used. Secondly, as already mentioned in the earlier part of this section, due to the limited achievable performance of the current classifier, the importance estimates from PIEE are unlikely to conclusively represent the significance within the data as PIEE is only an extension that enables importance estimate from a NN.

### Verifying Importance via Ablation Study

When the ground truth and existing domain knowledge are unavailable or limited, ablation study verification methods, such as Sensitivity Analysis^25^ and Reduce and Retrain^26^, can serve as a form of verification. Retrain with LOO and Singleton subsets is an ablation study which combines the principles of Sensitivity Analysis with Reduce and Retrain. It systematically retrains with LOO and Singleton subsets to utilise the deviation in performances achieved by the subsets to conclude feature importance for verification. Retrain with LOO and Singleton subsets is detailed in Section Methods for Comparison. For E3: Occupancy Detection and E4: HHAR, it was feasible to perform Retrain with LOO and Singleton because they only have 5 and 6 data channels respectively when feature importance is performed upon the data channels. This is ideal as the overhead is manageable.

In the case of E3: Occupancy Detection and E4: HHAR, they are both multivariate time series from the real world. This means that estimating feature importance would require the use of the adapted pairwise layer documented in Section Pairwise Importance Estimate Extension (PIEE) with Time Series, which can be analysed in 2 different ways, element-wise multiplication (EWM) upon the data channels, $\mathbf {X \odot C}$ where $\textbf{X}$ is an input with dimension $t \times c$ and $\textbf{C}$ is a vector variable with length *c*. Or the Hadamard product, $\mathbf {X \odot P}$, where both $\textbf{X}$ and $\textbf{P}$ are of the same dimension $t \times c$. Since the sliding window approach was used to process the time series into inputs of the corresponding NNs, the setup of the pairwise layer from the NNs are only limited to processing sliced windows of the time series instead of processing the whole time series. In this case, the Hadamard product $\mathbf {X \odot P}$ remains true, however the dimension will no longer be $t \times c$ but $t' \times c$ where $t'<t$ and $t'$ is defined by the sliding window’s setup. This reduces the effectiveness of the pairwise layer’s time step importance estimate, as the pairwise layer modification can no longer capture all aspects of the time series. However, information can still be extracted from the sliced windows via AHP and be used for approximation of the data channels’ importance. AHP was mentioned previously in Section Pairwise Importance Estimate Extension (PIEE) with Time Series.

**Fig. 19:**
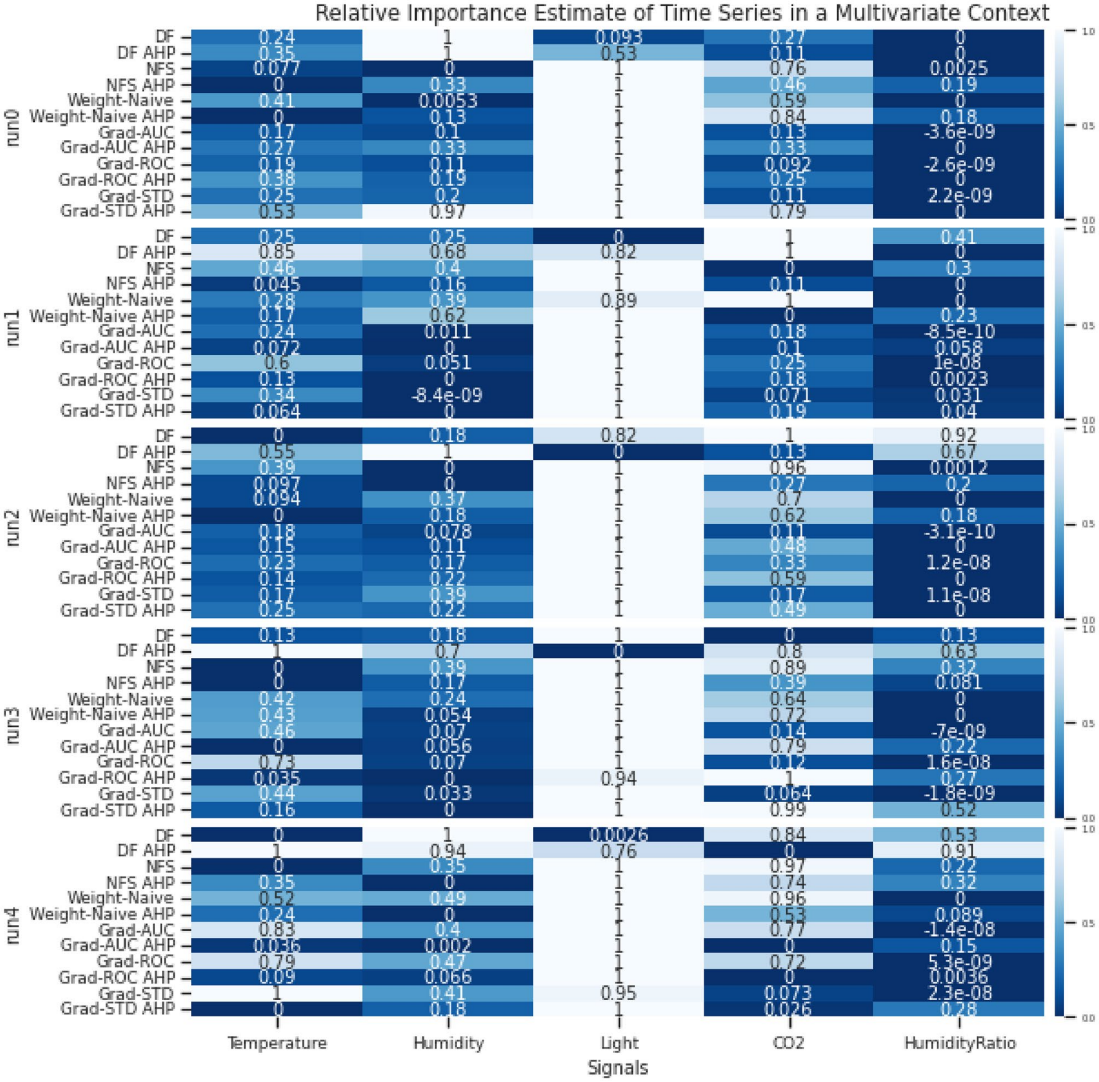
Occupancy Detection dataset with adapted existing embedded FS NN’s and PIEE’s importance estimate using EWM and AHP across 5 runs with different splits: Apart from DF, all methods using both approaches agree on Light being an important channel.

In experiment E3: Occupancy Detection of Section, Retrain with LOO and Singleton subsets were conducted for the corresponding 5 features: *Temperature*, *Humidity*, *Humidity ratio*, *Light* and $CO_2$. The results of Table 2 suggests *Light* to be the most important feature, because it has the lowest F1 Score during LOO, i.e when *Light* is left out of the input, and the highest F1 Score during Singleton, i.e when it is the only feature used as the input. This means the model suffered the most when it was not available, and the model was able to utilise its information effectively when it was the only one available. This suggests *Light* to be the most impactful among the features and is closely followed by $CO_2$.

This is empirically supported by the time series of each data channel, which is available in Fig. 8. The time series of each feature is presented and overlaid with a red and white background, where white indicates periods of time where the label for the binary classification is 1, and vice versa. Although the ground truth is not present, the representations of the signals also support the conclusion from Sensitivity Analysis using LOO and Singleton subsets, because a spike pattern can be identified between the *Light* and $CO_2$ signals and the labels.

Regarding the approach of EWM upon the data channels for E3: Occupancy Detection, every method, apart from the existing embedded FS NN method DF, agrees conclusively across the different runs with different train and validation split that *Light* is the most important data channel. Interestingly, both embedded FS NN method, NFS, and PIEE’s Weight-Naive support that $CO_2$ is also high in importance, whereas the *Gradient based analysis *methods do not support the same notion. A possible explanation could be that the models learned to over rely on the *Light* channel, therefore the response from the gradient is not noticeable in *Gradient based analysis*. Notably, in the AHP approach, all methods except DF agrees that *Light* is the most important feature again. Interestingly, *Gradient based analysis *methods would occasionally give higher importance to $CO_2$ although not conclusively across the runs. These findings are demonstrated in Fig. 19.

For Section E4: HHAR, Retrain with LOO and Singleton subsets were conducted for the corresponding 6 features: $accel_x$, $accel_y$, $accel_z$, $gyro_x$, $gyro_y$ and $gyro_z$. The results of Table 3 suggests $accel_x$ to be the most important feature, because it has the lowest F1 Score when during LOO, i.e when $accel_x$ is left out of the input, and the highest F1 Score during Singleton, i.e when it is the only feature used as the input. This means the model suffered the most when it was not available, and the model was able to utilise its information effectively when it was the only one available. This suggests $accel_x$ to be the most impactful among the features. Unlike previously, the other results of LOO and Singleton subsets are not conclusive enough for any other features to claim significance.

**Fig. 20:**
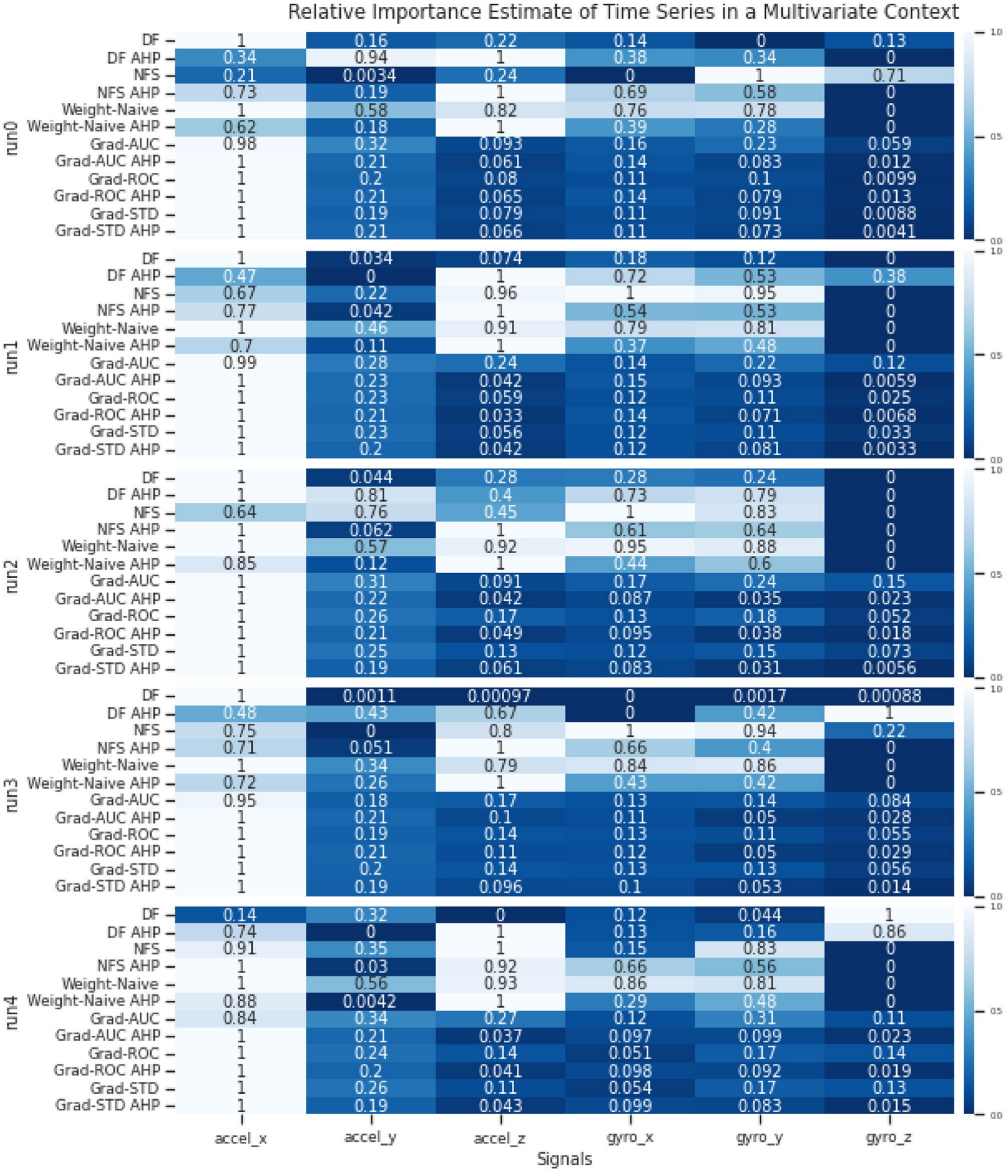
HHAR dataset with adapted existing embedded FS NN’s and PIEE’s importance estimate using EWM and AHP across 5 runs with different splits: Both approaches of PIEE’s Grad methods agree consistently.

Regarding the approach of EWM upon the data channels for E4: HHAR, both existing embedded FS NN methods, DF and NFS, are inconclusive across the different runs with different train and validation splits. This is especially prominent in NFS. For PIEE’s Grad methods and PIEE’s Weight-Naive, they all agree that $accel_x$ is the most important feature. However, Weight-Naive suggests that $accel_z$, $gyro_x$ and $gyro_y$ to be also of high importance, whereas this is not supported in the *Gradient based analysis *methods. The results are available in Fig. 20. Overall, Retrain with LOO and Singleton subsets reinforce that $accel_x$ to be the most important feature, however no further claims of importance can be made for the other features. In that respect, *Gradient based analysis *methods’ estimate are supported whilst Weight-Naive’s estimate cannot be fully verified. However, based on previous results from Section Verifying Importance via Ground Truth, they suggest Weight-Naive’s estimate to be highly susceptible to noise, therefore its estimate should be treated with caution. Notably, in the AHP approach, DF is inconclusive yet again across all runs, whereas although NFS and Weight-Naive are conclusive, their estimates are not inline with the conclusion of Retrain with LOO and Singleton subsets. Specifically NFS and Weight-Naive estimate $accel_z$ to be the most important and $accel_x$ to be secondary in most cases. This is contrary to $accel_x$ being the most important from what was established in Retrain with LOO and Singleton subsets. On the other hand, *Gradient based analysis *methods are conclusive across the different runs and estimate $accel_x$ to be the most important, which is inline with what was established. These findings are available in Fig. 20.

**Table 2:** F1-Scores of *LOO* and *Singleton* support that **Light** is the most important feature. This is followed by **CO2**.

**Method**	**Temperature**	**Humidity**	**Light**	**CO2**	**Humidity Ratio**
**LOO**	0.9831	0.9831	**0.9432**	*0.9829*	0.9833
**Singleton**	0.7131	0.4411	**0.9814**	*0.9305*	0.5527

**Table 3:** F1-Scores of *LOO* and *Singleton* support that **accel_x** to be the most important feature.

**Method**	**accel_x**	**accel_y**	**accel_z**	**gyro_x**	**gyro_y**	**gyro_z**
**LOO**	**0.6187**	0.7633	0.7363	0.7448	0.7660	0.7485
**Singleton**	**0.7633**	0.6187	0.7448	0.7660	0.7485	0.7363

### Performance Comparison

This part details the secondary focus of the experiments, which is the comparison of performance between the existing methods and the PIEE approach. The performance in question specifically refers to the F1-Score of the respective models for each approach where their importance estimates were derived from.

Regarding the simulated univariate time series and the simulated multivariate time series of E1: Simulated Time Series Datasets, there is no difference between the NN’s performance from the different existing embedded FS NN approaches, such as DF and NFS, and PIEE’s Weight-Naive and *Gradient based analysis *methods. This is due to the simplicity of the classification problem which we intended in order to have a clear ground truth for verification. This is the case for the simulated univariate time series, and the simulated multivariate time series.

**Table 4:** Performance (F1 Score) comparison between the adapted existing embedded NN FS approaches and the adapted PIEE’s methods with the AR EEG dataset, the Occupancy Detection dataset and the HHAR dataset, along with the Paired T Test results between the Baseline and the respective approaches. Bonferroni correction was used to account for multiple comparisons and the null hypothesis is rejected for $\alpha < (0.05 / 6)$. Holm correction was also used as an alternative to correct p-values for multiple comparisons. *AHP* signifies the approach of $\mathbf {X \odot P}$ was used. Otherwise, the EWM upon the data channels’s approach $\mathbf {X \odot C}$ was used.

	AR EEG				Occupancy Detection				HHAR			
	F1-score	t(5)	p-value	holm	F1-score	t(5)	p-value	holm	F1-score	t(5)	p-value	holm
**Baseline**	0.340	-	-	-	0.983	-	-	-	0.775	-	-	-
DF	0.129	*	*	*	0.549	*	*	*	0.491	*	*	*
DF AHP	0.158	*	*	*	0.441	*	*	*	0.058	*	*	*
NFS	0.293	1.306	0.200	0.999	0.984	0.189	0.855	1.0	0.704	−3.123	0.002	0.014
NFS AHP	0.245	2.434	0.020	0.120	0.975	−1.807	0.142	0.855	0.708	−2.965	0.004	0.020
Weight-Naive	0.320	0.513	0.611	1.0	0.984	0.467	0.653	1.0	0.768	0.284	0.777	1.0
Weight-Naive	0.329	0.304	0.763	1.0	0.983	−0.824	0.434	1.0	0.777	0.120	0.905	1.0
AHP												
Grad	0.305	0.917	0.365	1.0	0.984	0.544	0.601	1.0	0.770	0.211	0.834	1.0
Grad AHP	0.310	0.796	0.431	1.0	0.982	−1.014	0.340	1.0	0.776	0.045	0.965	1.0

For the datasets, E2: AR EEG Error Decoding, E3: Occupancy Detection, and E4: HHAR, the performances achieved by the NNs of different approaches and different adaptations are documented in Table 4. The Baseline denotes the original performance achieved by the functioning NN for each dataset before using any of the existing embedded FS NN approaches or PIEE’s methods. It is evident that the existing embedded FS NN approach, DF, suffers greatly in performance compared to the other approaches. This is contributed by the difficulty of fine tuning its parameters. An exhaustive search was conducted via Optuna^100^ for an experimental train and validation split, however the same parameters do not generalise well for the rest of the datasets. Therefore, the default parameters as suggested by Tensorflow^101^’s Keras framework was used as the final resort. The sub-optimal results explain why the estimate of features from Section Verifying Importance via Ablation Study are always inconclusive and unsupported. This reinforces how difficult it can be to fine tune the adaptation necessary for embedded NN methods to be applicable with time series, and DF is already a more stable variant of embedded methods that use regularisation

For the datasets of E2: AR EEG Error Decoding and E4: HHAR, the results from the Table 4 support that the adapted PIEE methods all performed similar to the baseline, however the adpated embedded FS NN approach, NFS, actually performed worse than the baseline. This is also supported by the Paired T-tests and p-value corrections. This supports the ablation study via removal of embedded architecture from Section Methods for Comparison. The results are the average of 5 repeated user-fold cross validation’s F1-Scores achieved by each respective method’s NN. User-fold cross validation is the equivalent of K fold cross validation where K is the number of participants within the study. This is detailed and explained in Section E2: AR EEG Error Decoding and Section E4: HHAR..

For the dataset of E3: Occupancy Detection, the results from the Table 4 support that the adapted existing embedded FS NN approach, NFS, and the adapted PIEE methods all performed similarly to the baseline. This is supported by Paired T-Tests and p-value corrections. The results are the average F1-Scores achieved by each respective method’s NN with 5 different train and validation splits. This supports the ablation study via removal of embedded architecture from Section Methods for Comparison.

## Discussion

The research questions, posed previously in Section Method, have been addressed by the findings from the experiments.

**RQ1: Can existing embedded FS NN approaches and PIEE identify importance within univariate time series? Do they agree with the results of xAI?** We have introduced our previous approach, PIEE^22^, in Section Pairwise Importance Estimate Extension (PIEE) with Time Series, where we established how the approach works in Fig. 3, and explained how the analysis of Importance Estiamte via Weight Profiles and Importance Estimate via Gradient Profiles can be conducted to procure importance estimates.

Based on the results from Section Verifying Importance via Ground Truth of the univariate time series from E1: Simulated Time Series Datasets, they demonstrated that existing embedded FS NN approaches, DF and NFS, are not compatible with identifying importance within univariate time series data. Conversely, PIEE’s *Gradient based analyses* has managed to consistently identify the important characteristics across the different runs to differentiate the classes in the classification tasks for the simulated univariate time series. Moreover, we further validated this against an established xAI method, DeepLIFT^27^. The results are available from Figs. 9, 10(a), and (b).

**RQ2: Can existing adapted embedded FS NN approaches and the adapated PIEE identify importance (i.e time steps and data channels) within multivariate time series?** We have further explained in Section Pairwise Importance Estimate Extension (PIEE) with Time Series what adaptation is required for multivariate time series, and demonstrated 2 variations of the pairwise layer adaptation setup as well as their associated analysis.

Based on the results from Section Verifying Importance via Ground Truth of the multivariate time series from E1: Simulated Time Series Datasets, they show that existing embedded FS NN approaches, DF and NFS, are also not compatible with identifying importance within multivariate time series data. However, similar to the findings from **RQ1**, PIEE’s *Gradient based analyses* have managed to consistently identify the important characteristics across the different runs to differentiate each class in the classification task for the simulated multivariate time series. These findings are verified by the ground truth and they are available from Figs. 13, 14 and 15. The same also holds true for a real-world physiological dataset concerned with error decoding, E2: AR EEG Error Decoding, where Section Verifying Importance via Existing Domain Knowledge demonstrated that PIEE’s *Gradient based analyses* can identify significance within the time steps and EEG channels inline with what has been established from existing literatures^83,89,90^. This is evident from Figs. 16 and 18 respectively. When compared with DeepLIFT, PIEE’s Gradient based analyses have also shown to be in agreement with DeepLIFT’s importance estimate in Figs. 16 and 17. Additionally, in Section Verifying Importance via Ablation Study, Gradient Profile analyses from E3: Occupancy Detection and E4: HHAR, demonstrated by Figs. 19 and 20, have managed to consistently agree with the results of the ablation study, Retrain with LOO and Singleton subsets, available from Tables 2 and 3.

**RQ3: Does the adaptation towards existing embedded NN approaches and PIEE negatively affect performance?** Based on the results from Section Performance Comparison, they indicate that the adapted PIEE’s methods, Weight-Naive from *Weight based analysis* and *Gradient based analysis* methods, do not negatively affect performance of the NN when compared to the baseline. On the other hand, existing embedded FS NN approaches, such as DF and NFS, have mixed results regarding the performance of the NN when compared to the baseline.

**Strengths and limitations** of the adapted approach: Pairwise Importance Estimate Extension (PIEE) with Time Series, has been observed throughout the experiments, and they are hereby discussed. The strength of the method includes:

- **Minimal complexity within the setup** Similar to the original PIEE’s approach, the setup introduces minimal external mechanism, it only requires a one-to-one multiplication between the PIEE and the input features, which is trivial to set up whilst allowing information of significance to be extracted from the input features. This makes the proposed adaptation easily deployable without the need to conduct any fine tuning of existing parameters, nor is there a need to fine tune any new parameters. This is an advantage over most existing deep learning feature selection methods^61,63^, as most of them require new mechanisms, which in turn calls for the fine tuning of parameters that is dependent on the dataset. This introduces additional cost of time and labour even though it could still result in subpar performance as shown by the experiments’ results. Also, compared to post-hoc explainabilty methods such as DeepLIFT^27^ or Layer-Wise Relevance Propagation^47^, the feature importance calculation is obtained here for *free*, as it is obtained as a by-product during training.
- **Accessible information** The minimal complexity from PIEE allows for information of significance to be easily accessible. The linear transformation allows for information to be extracted in a straightforward manner, otherwise such information would have been obfuscated behind the non-linearity workings of NNs. Particularly, with the complexity of RNNs, it would make the analysis of each feature from the backpropogation harder. Similar to before, the advantage of PIEE lies in its ease of integration into functioning architectures, as well as its ease of integration in terms of its analysis using informational profiles.
- **Higher consistency by combining statistical methods with NN’s internal information** Similar to what was established in PIEE, the results showed that the existing embedded FS NN methods are unable to estimate importance consistently nor correctly according to the ground truth, and the ablation study using Retrain with LOO and Singleton subsets. Whereas the adapted Gradient Profile analysis of PIEE continue to achieve high consistency across datasets, which can be verified by the ground truth and agrees with the results of Retrain with LOO and Singleton subsets.
- **Alternate workaround for embedded methods** Embedded FS NN methods usually cannot process multivariate time series. The deployment of embedded NN methods onto time series is scarce, and often difficult due to the incompatability with the data structure and non-linearity of time series. However, the adaptations introduced and explored within this work, i.e, the 2 variations of the pairwise layer adaptation setups have offered empirical insights into the process of how embedded NN methods can be improved to achieve consistent results with time series.
- **Interpretable time step importance within the context of time series** As mentioned previously, the application of embedded FS NN methods onto time series is scarce. Additionally, it is often difficult to distinguish the importance of the time steps respective to each time series in a multivariate time series context. The proposed adaptation offers a way to enable the estimate of importance for the time steps with respect to each time series via the Hadamard product approach, which has achieved consistent results throughout the experiments within this work, and they can also identify time step importance with respect to each time series.

The limitations of the method include:

- **Quality of importance estimate is dependent on the NN performance** Similar to the original PIEE’s approach, the proposed adaptation is limited by the achievable performance of the model that the extension attaches to. When the performance of the existing model is sub-optimal, the proposed methods cannot reliably extract useful information regarding the importance of features. This dependency is inherent to the setup of the approach as well as existing embedded NN methods, where the effect can be minimised but never be fully rid of.
- **Subjective interpretation that is dependent on the NN** Another similarity that is inherited from PIEE is that the proposed adaptation is limited by the internal mechanisms of the extended model. The PIEE approach would reflect what each model learned to rely on subjectively, rather than being able to identify feature importance objectively. Although this point is similar to the previous critique, it is different because this is not only subjected by NN’s performance. It can also be affected by the NN’s architecture. This is not something that is explored within this work, however performance difference between different architectures, and even between different amount of layers, does exists in literature^102,103^.
- **Interpretation relies on internal NN assumptions** The interpretation of the importance estimate is still subject to underlying NN assumptions of the internal mechanism, which is countered by *Gradient based analysis* that takes the fluctuation of gradient changes over time into account to offer a more grounded interpretation.
- **Approximation** PIEE is still only capable of providing an estimate of feature importance rather than a definitive measure. This limits the model from use cases where precision and accuracy are an absolute necessity.
- **Limited usefulness with trained models** From an application standpoint, in cases where trained models are readily available, the PIEE approach of an extension that collects information during training is irrelevant since training has already been done. In such cases, explainable AI post-hoc methods are more applicable and practical.

## Conclusion

To conclude, we have taken the previous approach of Pairwise Importance Estimate Extension^22^, PIEE, which offers different approaches toward analysing feature importance through the use of a pairwise layer. And we have adapted it within this work to analyse importance within the context of univariate and multivariate time series. Based on the foundation of PIEE and the existing embedded FS NN methods, we built upon PIEE with adaptations that allow for the estimate of importance within the context of time series. More specifically, the estimate of importance with multivariate time series, which consists of 2 dimensional data. Within Section Pairwise Importance Estimate Extension (PIEE) with Time Series, we detailed the adaptations, which revolves around 2 variations of the pairwise setups: Element-Wise Multiplication upon the data channels of a multivariate time series, or the Hadarmard Product, which is an element-wise multiplication between 2 matrices of the same dimensions. The estimate of importance within both univariate and multivariate time series context have been verified with multiple experiments, such as E1: Simulated Time Series Datasets, E2: AR EEG Error Decoding, E3: Occupancy Detection and E4: HHAR. In the experiments, the adapted PIEE’s methods were compared with existing adapted embedded FS NN methods, such as DF and NFS. DeepLIFT^27^, an established xAI post hoc method was also used for comparison when applicable. In Section Verifying Importance via Ground Truth, comparisons were made with the ground truth. In Section Verifying Importance via Existing Domain Knowledge, comparisons were made with existing domain knowledge. Lastly, in Section Verifying Importance via Ablation Study, they were verified with Retrain with LOO and Singleton subsets, a method focused on the ablation of feature subsets. The experiments concluded that *Gradient based analysis*’s estimate of importance are consistently better than existing embedded NN methods in both univariate and multivariate time series context. In Section Performance Comparison, it was also found that the adapted PIEE’s methods do not negatively affect performance when compared to the baseline model with the ablation of any embedded architecture. Based on the results achieved within this work, we answered the posed research questions. We showed PIEE to be capable of estimating importance within an univariate time series context. We also showed that PIEE can be adapated to estimate importance within a multivariate time series context. This is significant as there exist little embedded NN methods that can be applied to the context of time series, and there exists even less embedded NN methods that can produce conclusive results. 

Furthermore, we examined the strengths and limitations of the adapted PIEE approach. In terms of strengths, the proposed adapted PIEE retains majority of the strengths from the original approach. It offers a generalisable mechanism with minimal complexity where the importance estimate is accessible and has high consistency across the different datasets. In the future, we aim to apply the adapted PIEE approach with more time series datasets in both univariate and multivariate contexts to investigate the consistency and interpretation of the importance estimate, especially in terms of relative time step importance within a time series. We hope other researchers will find our research useful, and make use of our method in their work.

## Supplementary Information

Supplementary Information.

## Data Availability

The data used in the experiments of this work and their respective data repository have been cited in the relevant sections. The Git repository of the scripts used in the experiments is available at https://github.com/thehcclab/pairwise-importance-estimate-extension-with-time-series-adaptation. Inquires regarding the data can be sent to the author, Ho Tung Jeremy Chan.
