# Supplementary material for: Explainability and importance estimate of time series classifier via embedded neural network
Source: Sci Rep. 2025 Oct 3;15:34515. doi: 10.1038/s41598-025-17703-w (PMC12494753; doi:10.1038/s41598-025-17703-w)
Supplement: Supplementary file 1 — Supplementary Information. [file 41598_2025_17703_MOESM1_ESM.pdf]

Explainability and Importance Estimate of Time Series Classifier via Embedded Neural Network

Ho Tung Jeremy Chan[1,2,*,+], Ilija Šimić[2], and Eduardo Veas[1,2,+]

[1]Interactive System and Data Science, Graz University of Technology, Graz, 8010, Austria
[2]Human AI Interaction, Know-Center GmbH, Graz, 8010, Austria
[*]jchan@know-center.at
[+]these authors contributed equally to this work

ABSTRACT

Time series is common across disciplines, however the analysis of time series is not trivial due to inter- and intra-relationships between ordered data sequences. This imposes limitation upon the interpretation and importance estimate of the features within a time series. In the case of multivariate time series, these features are the individual time series and the time steps, which are intertwined. There exist many time series analyses, such as Autocorrelation and Granger Causality, which are based on statistic or econometric approaches. However analyses that can inform the importance of features within a time series are uncommon, especially with methods that utilise embedded methods of neural network (NN). We approach this problem by expanding upon our previous work, Pairwise Importance Estimate Extension (PIEE). We made adaptations toward the existing method to make it compatible with time series. This led to the formulation of aggregated Hadamard product, which can produce an importance estimate for each time point within a multivariate time series. This subsequently allows each time series within a multivariate time series to be interpreted as well. Within this work, we conducted an empirical study with univariate and multivariate time series, where we compared interpretation and importance estimate of features from existing embedded NN approaches, an explainable AI (xAI) approach, and our adapted PIEE approach. We verified interpretation and importance estimate via ground truth or existing domain knowledge when it is available. Otherwise, we conducted an ablation study by retraining the model with Leave-One-Out and Singleton feature subsets to see their contribution towards model performance. Our adapted PIEE method was able to produce various feature importance heatmaps and rankings inline with the ground truth, the existing domain knowledge or the ablation study.

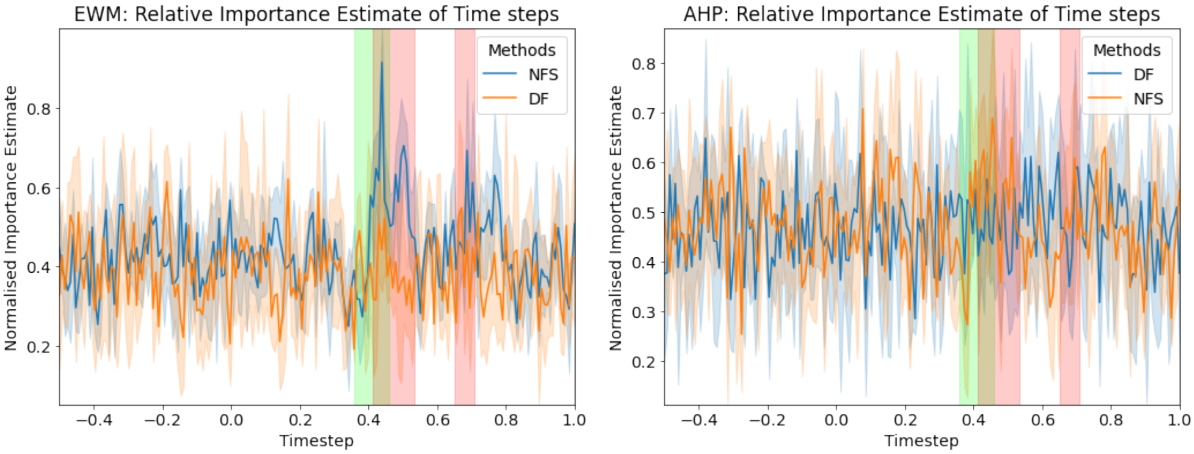

Figure S1. AR EEG dataset with adapted existing embedded FS NN's importance estimate of time steps using EWM (left) and AHP (right) across 5 runs of user-fold split: red highlights periods with significant difference, green highlights event related theta peak.

The graphs demonstrates that both adaptations of DF cannot produce meaningful results. As for NFS, although its EWM adaptation are able to have some responses during the significant time periods, its AHP adaption also cannot produce meaningful results.

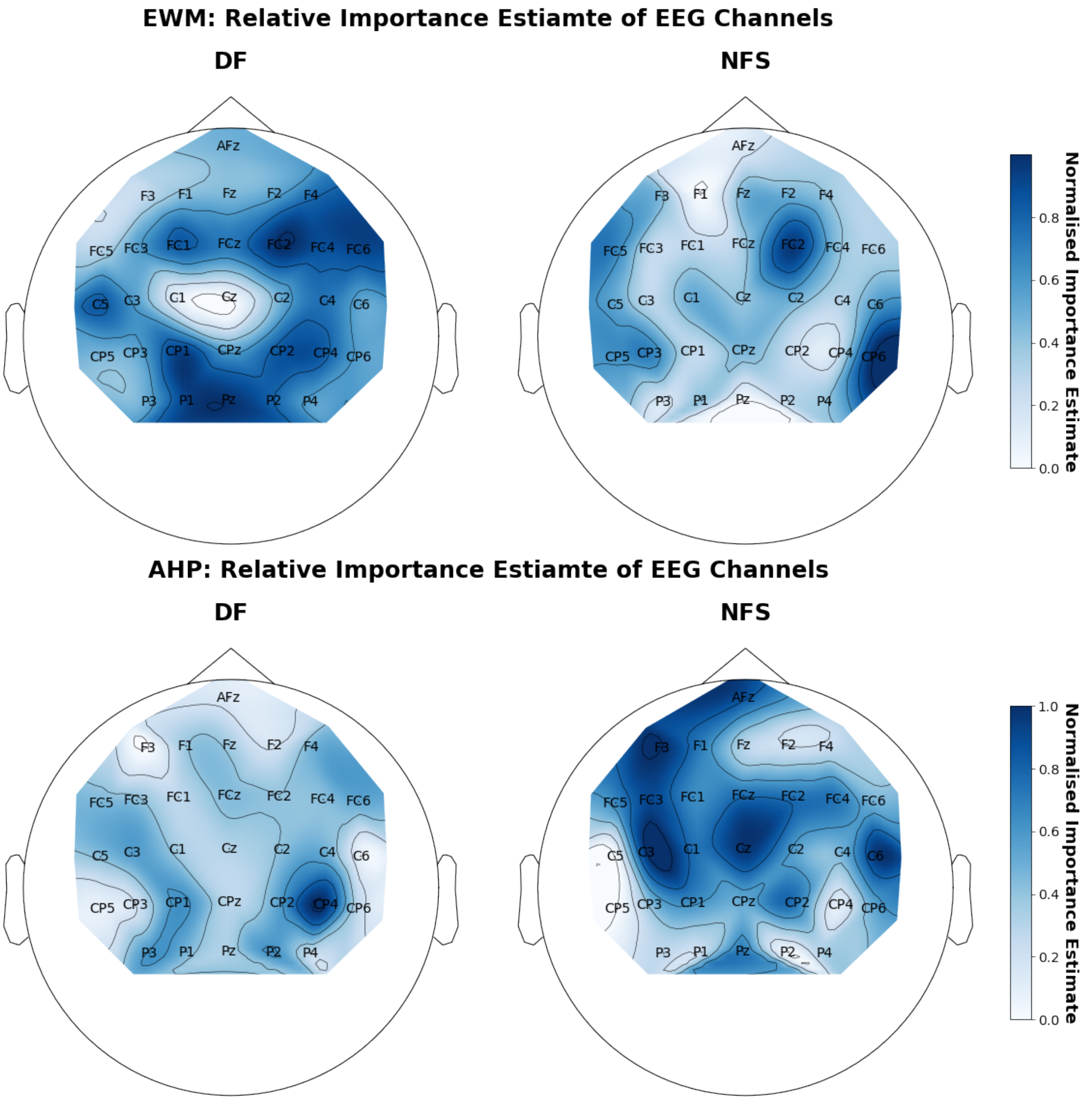

Figure S2. AR EEG dataset with adapted existing embedded FS NN's importance estimate of EEG channels using EWM (left) and AHP (right) across 5 runs of user-fold split: the dark blue areas represent higher importance estimate than lighter blue areas, as informed by the colour bars.

The graphs show that both DF and NFS are inconclusive and nonsenical across both adaptations.
